# Search for direct pair production of supersymmetric partners to the ${\uptau }_{}^{}$ lepton in proton–proton collisions at $\sqrt{s}=13\,\text {TeV}$

**DOI:** 10.1140/epjc/s10052-020-7739-7

**Published:** 2020-03-02

**Authors:** The CMS Collaboration, A. M. Sirunyan, A. Tumasyan, W. Adam, F. Ambrogi, T. Bergauer, J. Brandstetter, M. Dragicevic, J. Erö, A. Escalante Del Valle, M. Flechl, R. Frühwirth, M. Jeitler, N. Krammer, I. Krätschmer, D. Liko, T. Madlener, I. Mikulec, N. Rad, J. Schieck, R. Schöfbeck, M. Spanring, D. Spitzbart, W. Waltenberger, C.-E. Wulz, M. Zarucki, V. Drugakov, V. Mossolov, J. Suarez Gonzalez, M. R. Darwish, E. A. De Wolf, D. Di Croce, X. Janssen, A. Lelek, M. Pieters, H. Rejeb Sfar, H. Van Haevermaet, P. Van Mechelen, S. Van Putte, N. Van Remortel, F. Blekman, E. S. Bols, S. S. Chhibra, J. D’Hondt, J. De Clercq, D. Lontkovskyi, S. Lowette, I. Marchesini, S. Moortgat, L. Moreels, Q. Python, K. Skovpen, S. Tavernier, W. Van Doninck, P. Van Mulders, I. Van Parijs, D. Beghin, B. Bilin, H. Brun, B. Clerbaux, G. De Lentdecker, H. Delannoy, B. Dorney, L. Favart, A. Grebenyuk, A. K. Kalsi, A. Popov, N. Postiau, E. Starling, L. Thomas, C. Vander Velde, P. Vanlaer, D. Vannerom, T. Cornelis, D. Dobur, I. Khvastunov, M. Niedziela, C. Roskas, D. Trocino, M. Tytgat, W. Verbeke, B. Vermassen, M. Vit, N. Zaganidis, O. Bondu, G. Bruno, C. Caputo, P. David, C. Delaere, M. Delcourt, A. Giammanco, V. Lemaitre, A. Magitteri, J. Prisciandaro, A. Saggio, M. Vidal Marono, P. Vischia, J. Zobec, F. L. Alves, G. A. Alves, G. Correia Silva, C. Hensel, A. Moraes, P. Rebello Teles, E. Belchior Batista Das Chagas, W. Carvalho, J. Chinellato, E. Coelho, E. M. Da Costa, G. G. Da Silveira, D. De Jesus Damiao, C. De Oliveira Martins, S. Fonseca De Souza, L. M. Huertas Guativa, H. Malbouisson, J. Martins, D. Matos Figueiredo, M. Medina Jaime, M. Melo De Almeida, C. Mora Herrera, L. Mundim, H. Nogima, W. L. Prado Da Silva, L. J. Sanchez Rosas, A. Santoro, A. Sznajder, M. Thiel, E. J. Tonelli Manganote, F. Torres Da Silva De Araujo, A. Vilela Pereira, C. A. Bernardes, L. Calligaris, T. R. Fernandez Perez Tomei, E. M. Gregores, D. S. Lemos, P. G. Mercadante, S. F. Novaes, SandraS. Padula, A. Aleksandrov, G. Antchev, R. Hadjiiska, P. Iaydjiev, A. Marinov, M. Misheva, M. Rodozov, M. Shopova, G. Sultanov, M. Bonchev, A. Dimitrov, T. Ivanov, L. Litov, B. Pavlov, P. Petkov, W. Fang, X. Gao, L. Yuan, M. Ahmad, G. M. Chen, H. S. Chen, M. Chen, C. H. Jiang, D. Leggat, H. Liao, Z. Liu, S. M. Shaheen, A. Spiezia, J. Tao, E. Yazgan, H. Zhang, S. Zhang, J. Zhao, A. Agapitos, Y. Ban, G. Chen, A. Levin, J. Li, L. Li, Q. Li, Y. Mao, S. J. Qian, D. Wang, Q. Wang, Z. Hu, Y. Wang, C. Avila, A. Cabrera, L. F. Chaparro Sierra, C. Florez, C. F. González Hernández, M. A. Segura Delgado, J. Mejia Guisao, J. D. Ruiz Alvarez, C. A. Salazar González, N. Vanegas Arbelaez, D. Giljanović, N. Godinovic, D. Lelas, I. Puljak, T. Sculac, Z. Antunovic, M. Kovac, V. Brigljevic, S. Ceci, D. Ferencek, K. Kadija, B. Mesic, M. Roguljic, A. Starodumov, T. Susa, M. W. Ather, A. Attikis, E. Erodotou, A. Ioannou, M. Kolosova, S. Konstantinou, G. Mavromanolakis, J. Mousa, C. Nicolaou, F. Ptochos, P. A. Razis, H. Rykaczewski, D. Tsiakkouri, M. Finger, M. Finger, A. Kveton, J. Tomsa, E. Ayala, E. Carrera Jarrin, S. Abu Zeid, S. Khalil, S. Bhowmik, A. Carvalho Antunes De Oliveira, R. K. Dewanjee, K. Ehataht, M. Kadastik, M. Raidal, C. Veelken, P. Eerola, L. Forthomme, H. Kirschenmann, K. Osterberg, M. Voutilainen, F. Garcia, J. Havukainen, J. K. Heikkilä, T. Järvinen, V. Karimäki, R. Kinnunen, T. Lampén, K. Lassila-Perini, S. Laurila, S. Lehti, T. Lindén, P. Luukka, T. Mäenpää, H. Siikonen, E. Tuominen, J. Tuominiemi, T. Tuuva, M. Besancon, F. Couderc, M. Dejardin, D. Denegri, B. Fabbro, J. L. Faure, F. Ferri, S. Ganjour, A. Givernaud, P. Gras, G. Hamel de Monchenault, P. Jarry, C. Leloup, E. Locci, J. Malcles, J. Rander, A. Rosowsky, M. Ö. Sahin, A. Savoy-Navarro, M. Titov, S. Ahuja, C. Amendola, F. Beaudette, P. Busson, C. Charlot, B. Diab, G. Falmagne, R. Granier de Cassagnac, I. Kucher, A. Lobanov, C. Martin Perez, M. Nguyen, C. Ochando, P. Paganini, J. Rembser, R. Salerno, J. B. Sauvan, Y. Sirois, A. Zabi, A. Zghiche, J.-L. Agram, J. Andrea, D. Bloch, G. Bourgatte, J.-M. Brom, E. C. Chabert, C. Collard, E. Conte, J.-C. Fontaine, D. Gelé, U. Goerlach, M. Jansová, A.-C. Le Bihan, N. Tonon, P. Van Hove, S. Gadrat, S. Beauceron, C. Bernet, G. Boudoul, C. Camen, N. Chanon, R. Chierici, D. Contardo, P. Depasse, H. El Mamouni, J. Fay, S. Gascon, M. Gouzevitch, B. Ille, Sa. Jain, F. Lagarde, I. B. Laktineh, H. Lattaud, M. Lethuillier, L. Mirabito, S. Perries, V. Sordini, G. Touquet, M. Vander Donckt, S. Viret, A. Khvedelidze, Z. Tsamalaidze, C. Autermann, L. Feld, M. K. Kiesel, K. Klein, M. Lipinski, D. Meuser, A. Pauls, M. Preuten, M. P. Rauch, C. Schomakers, J. Schulz, M. Teroerde, B. Wittmer, A. Albert, M. Erdmann, S. Erdweg, T. Esch, B. Fischer, R. Fischer, S. Ghosh, T. Hebbeker, K. Hoepfner, H. Keller, L. Mastrolorenzo, M. Merschmeyer, A. Meyer, P. Millet, G. Mocellin, S. Mondal, S. Mukherjee, D. Noll, A. Novak, T. Pook, A. Pozdnyakov, T. Quast, M. Radziej, Y. Rath, H. Reithler, M. Rieger, J. Roemer, A. Schmidt, S. C. Schuler, A. Sharma, S. Thüer, S. Wiedenbeck, G. Flügge, W. Haj Ahmad, O. Hlushchenko, T. Kress, T. Müller, A. Nehrkorn, A. Nowack, C. Pistone, O. Pooth, D. Roy, H. Sert, A. Stahl, M. Aldaya Martin, P. Asmuss, I. Babounikau, H. Bakhshiansohi, K. Beernaert, O. Behnke, U. Behrens, A. Bermúdez Martínez, D. Bertsche, A. A. Bin Anuar, K. Borras, V. Botta, A. Campbell, A. Cardini, P. Connor, S. Consuegra Rodríguez, C. Contreras-Campana, V. Danilov, A. De Wit, M. M. Defranchis, L. Didukh, C. Diez Pardos, D. Domínguez Damiani, G. Eckerlin, D. Eckstein, T. Eichhorn, A. Elwood, E. Eren, E. Gallo, A. Geiser, J. M. Grados Luyando, A. Grohsjean, M. Guthoff, M. Haranko, A. Harb, A. Jafari, N. Z. Jomhari, H. Jung, A. Kasem, M. Kasemann, H. Kaveh, J. Keaveney, C. Kleinwort, J. Knolle, D. Krücker, W. Lange, T. Lenz, J. Leonard, J. Lidrych, K. Lipka, W. Lohmann, R. Mankel, I.-A. Melzer-Pellmann, A. B. Meyer, M. Meyer, M. Missiroli, G. Mittag, J. Mnich, A. Mussgiller, V. Myronenko, D. Pérez Adán, S. K. Pflitsch, D. Pitzl, A. Raspereza, A. Saibel, M. Savitskyi, V. Scheurer, P. Schütze, C. Schwanenberger, R. Shevchenko, A. Singh, H. Tholen, O. Turkot, A. Vagnerini, M. Van De Klundert, G. P. Van Onsem, R. Walsh, Y. Wen, K. Wichmann, C. Wissing, O. Zenaiev, R. Zlebcik, R. Aggleton, S. Bein, L. Benato, A. Benecke, V. Blobel, T. Dreyer, A. Ebrahimi, A. Fröhlich, C. Garbers, E. Garutti, D. Gonzalez, P. Gunnellini, J. Haller, A. Hinzmann, A. Karavdina, G. Kasieczka, R. Klanner, R. Kogler, N. Kovalchuk, S. Kurz, V. Kutzner, J. Lange, T. Lange, A. Malara, J. Multhaup, C. E. N. Niemeyer, A. Perieanu, A. Reimers, O. Rieger, C. Scharf, P. Schleper, S. Schumann, J. Schwandt, J. Sonneveld, H. Stadie, G. Steinbrück, F. M. Stober, M. Stöver, B. Vormwald, I. Zoi, M. Akbiyik, C. Barth, M. Baselga, S. Baur, T. Berger, E. Butz, R. Caspart, T. Chwalek, W. De Boer, A. Dierlamm, K. El Morabit, N. Faltermann, M. Giffels, P. Goldenzweig, A. Gottmann, M. A. Harrendorf, F. Hartmann, U. Husemann, S. Kudella, S. Mitra, M. U. Mozer, Th. Müller, M. Musich, A. Nürnberg, G. Quast, K. Rabbertz, M. Schröder, I. Shvetsov, H. J. Simonis, R. Ulrich, M. Weber, C. Wöhrmann, R. Wolf, G. Anagnostou, P. Asenov, G. Daskalakis, T. Geralis, A. Kyriakis, D. Loukas, G. Paspalaki, M. Diamantopoulou, G. Karathanasis, P. Kontaxakis, A. Manousakis-katsikakis, A. Panagiotou, I. Papavergou, N. Saoulidou, A. Stakia, K. Theofilatos, K. Vellidis, E. Vourliotis, G. Bakas, K. Kousouris, I. Papakrivopoulos, G. Tsipolitis, I. Evangelou, C. Foudas, P. Gianneios, P. Katsoulis, P. Kokkas, S. Mallios, K. Manitara, N. Manthos, I. Papadopoulos, J. Strologas, F. A. Triantis, D. Tsitsonis, M. Bartók, M. Csanad, P. Major, K. Mandal, A. Mehta, M. I. Nagy, G. Pasztor, O. Surányi, G. I. Veres, G. Bencze, C. Hajdu, D. Horvath, F. Sikler, T. Vámi, V. Veszpremi, G. Vesztergombi, N. Beni, S. Czellar, J. Karancsi, A. Makovec, J. Molnar, Z. Szillasi, P. Raics, D. Teyssier, Z. L. Trocsanyi, B. Ujvari, T. Csorgo, W. J. Metzger, F. Nemes, T. Novak, S. Choudhury, J. R. Komaragiri, P. C. Tiwari, S. Bahinipati, C. Kar, G. Kole, P. Mal, V. K. Muraleedharan Nair Bindhu, A. Nayak, D. K. Sahoo, S. K. Swain, S. Bansal, S. B. Beri, V. Bhatnagar, S. Chauhan, R. Chawla, N. Dhingra, R. Gupta, A. Kaur, M. Kaur, S. Kaur, P. Kumari, M. Lohan, M. Meena, K. Sandeep, S. Sharma, J. B. Singh, A. K. Virdi, A. Bhardwaj, B. C. Choudhary, R. B. Garg, M. Gola, S. Keshri, Ashok Kumar, S. Malhotra, M. Naimuddin, P. Priyanka, K. Ranjan, Aashaq Shah, R. Sharma, R. Bhardwaj, M. Bharti, R. Bhattacharya, S. Bhattacharya, U. Bhawandeep, D. Bhowmik, S. Dey, S. Dutta, S. Ghosh, M. Maity, K. Mondal, S. Nandan, A. Purohit, P. K. Rout, G. Saha, S. Sarkar, T. Sarkar, M. Sharan, B. Singh, S. Thakur, P. K. Behera, P. Kalbhor, A. Muhammad, P. R. Pujahari, A. Sharma, A. K. Sikdar, R. Chudasama, D. Dutta, V. Jha, V. Kumar, D. K. Mishra, P. K. Netrakanti, L. M. Pant, P. Shukla, T. Aziz, M. A. Bhat, S. Dugad, G. B. Mohanty, N. Sur, RavindraKumar Verma, S. Banerjee, S. Bhattacharya, S. Chatterjee, P. Das, M. Guchait, S. Karmakar, S. Kumar, G. Majumder, K. Mazumdar, N. Sahoo, S. Sawant, S. Chauhan, S. Dube, V. Hegde, A. Kapoor, K. Kothekar, S. Pandey, A. Rane, A. Rastogi, S. Sharma, S. Chenarani, E. Eskandari Tadavani, S. M. Etesami, M. Khakzad, M. Mohammadi Najafabadi, M. Naseri, F. Rezaei Hosseinabadi, M. Felcini, M. Grunewald, M. Abbrescia, R. Aly, C. Calabria, A. Colaleo, D. Creanza, L. Cristella, N. De Filippis, M. De Palma, A. Di Florio, L. Fiore, A. Gelmi, G. Iaselli, M. Ince, S. Lezki, G. Maggi, M. Maggi, G. Miniello, S. My, S. Nuzzo, A. Pompili, G. Pugliese, R. Radogna, A. Ranieri, G. Selvaggi, L. Silvestris, R. Venditti, P. Verwilligen, G. Abbiendi, C. Battilana, D. Bonacorsi, L. Borgonovi, S. Braibant-Giacomelli, R. Campanini, P. Capiluppi, A. Castro, F. R. Cavallo, C. Ciocca, G. Codispoti, M. Cuffiani, G. M. Dallavalle, F. Fabbri, A. Fanfani, E. Fontanesi, P. Giacomelli, C. Grandi, L. Guiducci, F. Iemmi, S. Lo Meo, S. Marcellini, G. Masetti, F. L. Navarria, A. Perrotta, F. Primavera, A. M. Rossi, T. Rovelli, G. P. Siroli, N. Tosi, S. Albergo, S. Costa, A. Di Mattia, R. Potenza, A. Tricomi, C. Tuve, G. Barbagli, R. Ceccarelli, K. Chatterjee, V. Ciulli, C. Civinini, R. D’Alessandro, E. Focardi, G. Latino, P. Lenzi, M. Meschini, S. Paoletti, G. Sguazzoni, D. Strom, L. Viliani, L. Benussi, S. Bianco, D. Piccolo, M. Bozzo, F. Ferro, R. Mulargia, E. Robutti, S. Tosi, A. Benaglia, A. Beschi, F. Brivio, V. Ciriolo, S. Di Guida, M. E. Dinardo, P. Dini, S. Gennai, A. Ghezzi, P. Govoni, L. Guzzi, M. Malberti, S. Malvezzi, D. Menasce, F. Monti, L. Moroni, G. Ortona, M. Paganoni, D. Pedrini, S. Ragazzi, T. Tabarelli de Fatis, D. Zuolo, S. Buontempo, N. Cavallo, A. De Iorio, A. Di Crescenzo, F. Fabozzi, F. Fienga, G. Galati, A. O. M. Iorio, L. Lista, S. Meola, P. Paolucci, B. Rossi, C. Sciacca, E. Voevodina, P. Azzi, N. Bacchetta, D. D.Bisello, A. Boletti, A. Bragagnolo, R. Carlin, P. Checchia, P. De Castro Manzano, T. Dorigo, U. Dosselli, F. Gasparini, U. Gasparini, A. Gozzelino, S. Y. Hoh, P. Lujan, M. Margoni, A. T. Meneguzzo, J. Pazzini, M. Presilla, P. Ronchese, R. Rossin, F. Simonetto, A. Tiko, M. Tosi, M. Zanetti, P. Zotto, G. Zumerle, A. Braghieri, P. Montagna, S. P. Ratti, V. Re, M. Ressegotti, C. Riccardi, P. Salvini, I. Vai, P. Vitulo, M. Biasini, G. M. Bilei, C. Cecchi, D. Ciangottini, L. Fanò, P. Lariccia, R. Leonardi, E. Manoni, G. Mantovani, V. Mariani, M. Menichelli, A. Rossi, A. Santocchia, D. Spiga, K. Androsov, P. Azzurri, G. Bagliesi, V. Bertacchi, L. Bianchini, T. Boccali, R. Castaldi, M. A. Ciocci, R. Dell’Orso, G. Fedi, L. Giannini, A. Giassi, M. T. Grippo, F. Ligabue, E. Manca, G. Mandorli, A. Messineo, F. Palla, A. Rizzi, G. Rolandi, S. Roy Chowdhury, A. Scribano, P. Spagnolo, R. Tenchini, G. Tonelli, N. Turini, A. Venturi, P. G. Verdini, F. Cavallari, M. Cipriani, D. Del Re, E. Di Marco, M. Diemoz, E. Longo, B. Marzocchi, P. Meridiani, G. Organtini, F. Pandolfi, R. Paramatti, C. Quaranta, S. Rahatlou, C. Rovelli, F. Santanastasio, L. Soffi, N. Amapane, R. Arcidiacono, S. Argiro, M. Arneodo, N. Bartosik, R. Bellan, C. Biino, A. Cappati, N. Cartiglia, S. Cometti, M. Costa, R. Covarelli, N. Demaria, B. Kiani, C. Mariotti, S. Maselli, E. Migliore, V. Monaco, E. Monteil, M. Monteno, M. M. Obertino, L. Pacher, N. Pastrone, M. Pelliccioni, G. L. Pinna Angioni, A. Romero, M. Ruspa, R. Sacchi, R. Salvatico, V. Sola, A. Solano, D. Soldi, A. Staiano, S. Belforte, V. Candelise, M. Casarsa, F. Cossutti, A. Da Rold, G. Della Ricca, F. Vazzoler, A. Zanetti, B. Kim, D. H. Kim, G. N. Kim, M. S. Kim, J. Lee, S. W. Lee, C. S. Moon, Y. D. Oh, S. I. Pak, S. Sekmen, D. C. Son, Y. C. Yang, H. Kim, D. H. Moon, G. Oh, B. Francois, T. J. Kim, J. Park, S. Cho, S. Choi, Y. Go, D. Gyun, S. Ha, B. Hong, K. Lee, K. S. Lee, J. Lim, J. Park, S. K. Park, Y. Roh, J. Yoo, J. Goh, H. S. Kim, J. Almond, J. H. Bhyun, J. Choi, S. Jeon, J. Kim, J. S. Kim, H. Lee, K. Lee, S. Lee, K. Nam, M. Oh, S. B. Oh, B. C. Radburn-Smith, U. K. Yang, H. D. Yoo, I. Yoon, G. B. Yu, D. Jeon, H. Kim, J. H. Kim, J. S. H. Lee, I. C. Park, I. Watson, Y. Choi, C. Hwang, Y. Jeong, J. Lee, Y. Lee, I. Yu, V. Veckalns, V. Dudenas, A. Juodagalvis, G. Tamulaitis, J. Vaitkus, Z. A. Ibrahim, F. Mohamad Idris, W. A. T. Wan Abdullah, M. N. Yusli, Z. Zolkapli, J. F. Benitez, A. Castaneda Hernandez, J. A. Murillo Quijada, L. Valencia Palomo, H. Castilla-Valdez, E. De La Cruz-Burelo, I. Heredia-De La Cruz, R. Lopez-Fernandez, A. Sanchez-Hernandez, S. Carrillo Moreno, C. Oropeza Barrera, M. Ramirez-Garcia, F. Vazquez Valencia, J. Eysermans, I. Pedraza, H. A. Salazar Ibarguen, C. Uribe Estrada, A. Morelos Pineda, N. Raicevic, D. Krofcheck, S. Bheesette, P. H. Butler, A. Ahmad, M. Ahmad, Q. Hassan, H. R. Hoorani, W. A. Khan, M. A. Shah, M. Shoaib, M. Waqas, V. Avati, L. Grzanka, M. Malawski, H. Bialkowska, M. Bluj, B. Boimska, M. Górski, M. Kazana, M. Szleper, P. Zalewski, K. Bunkowski, A. Byszuk, K. Doroba, A. Kalinowski, M. Konecki, J. Krolikowski, M. Misiura, M. Olszewski, A. Pyskir, M. Walczak, M. Araujo, P. Bargassa, D. Bastos, A. Di Francesco, P. Faccioli, B. Galinhas, M. Gallinaro, J. Hollar, N. Leonardo, J. Seixas, K. Shchelina, G. Strong, O. Toldaiev, J. Varela, V. Alexakhin, A. Baginyan, M. Gavrilenko, I. Golutvin, I. Gorbunov, A. Kamenev, V. Karjavine, V. Korenkov, A. Lanev, A. Malakhov, V. Matveev, V. V. Mitsyn, P. Moisenz, V. Palichik, V. Perelygin, M. Savina, S. Shmatov, S. Shulha, V. Trofimov, A. Zarubin, L. Chtchipounov, V. Golovtsov, Y. Ivanov, V. Kim, E. Kuznetsova, P. Levchenko, V. Murzin, V. Oreshkin, I. Smirnov, D. Sosnov, V. Sulimov, L. Uvarov, A. Vorobyev, Yu. Andreev, A. Dermenev, S. Gninenko, N. Golubev, A. Karneyeu, M. Kirsanov, N. Krasnikov, A. Pashenkov, D. Tlisov, A. Toropin, V. Epshteyn, V. Gavrilov, N. Lychkovskaya, A. Nikitenko, V. Popov, I. Pozdnyakov, G. Safronov, A. Spiridonov, A. Stepennov, M. Toms, E. Vlasov, A. Zhokin, T. Aushev, M. Chadeeva, P. Parygin, D. Philippov, E. Popova, V. Rusinov, V. Andreev, M. Azarkin, I. Dremin, M. Kirakosyan, A. Terkulov, A. Baskakov, A. Belyaev, E. Boos, V. Bunichev, M. Dubinin, L. Dudko, A. Ershov, A. Gribushin, V. Klyukhin, O. Kodolova, I. Lokhtin, S. Obraztsov, V. Savrin, A. Barnyakov, V. Blinov, T. Dimova, L. Kardapoltsev, Y. Skovpen, I. Azhgirey, I. Bayshev, S. Bitioukov, V. Kachanov, D. Konstantinov, P. Mandrik, V. Petrov, R. Ryutin, S. Slabospitskii, A. Sobol, S. Troshin, N. Tyurin, A. Uzunian, A. Volkov, A. Babaev, A. Iuzhakov, V. Okhotnikov, V. Borchsh, V. Ivanchenko, E. Tcherniaev, P. Adzic, P. Cirkovic, D. Devetak, M. Dordevic, P. Milenovic, J. Milosevic, M. Stojanovic, M. Aguilar-Benitez, J. Alcaraz Maestre, A. lvarez Fernández, I. Bachiller, M. Barrio Luna, J. A. Brochero Cifuentes, C. A. Carrillo Montoya, M. Cepeda, M. Cerrada, N. Colino, B. De La Cruz, A. Delgado Peris, C. Fernandez Bedoya, J. P. Fernández Ramos, J. Flix, M. C. Fouz, O. Gonzalez Lopez, S. Goy Lopez, J. M. Hernandez, M. I. Josa, D. Moran, Navarro Tobar, A. Pérez-Calero Yzquierdo, J. Puerta Pelayo, I. Redondo, L. Romero, S. Sánchez Navas, M. S. Soares, A. Triossi, C. Willmott, C. Albajar, J. F. de Trocóniz, B. Alvarez Gonzalez, J. Cuevas, C. Erice, J. Fernandez Menendez, S. Folgueras, I. Gonzalez Caballero, J. R. González Fernández, E. Palencia Cortezon, V. Rodríguez Bouza, S. Sanchez Cruz, I. J. Cabrillo, A. Calderon, B. Chazin Quero, J. Duarte Campderros, M. Fernandez, P. J. Fernández Manteca, A. García Alonso, G. Gomez, C. Martinez Rivero, P. Martinez Ruiz del Arbol, F. Matorras, J. Piedra Gomez, C. Prieels, T. Rodrigo, A. Ruiz-Jimeno, L. Russo, L. Scodellaro, N. Trevisani, I. Vila, J. M. Vizan Garcia, K. Malagalage, W. G. D. Dharmaratna, N. Wickramage, D. Abbaneo, B. Akgun, E. Auffray, G. Auzinger, J. Baechler, P. Baillon, A. H. Ball, D. Barney, J. Bendavid, M. Bianco, A. Bocci, P. Bortignon, E. Bossini, C. Botta, E. Brondolin, T. Camporesi, A. Caratelli, G. Cerminara, E. Chapon, G. Cucciati, D. d’Enterria, A. Dabrowski, N. Daci, V. Daponte, A. David, O. Davignon, A. De Roeck, N. Deelen, M. Deile, M. Dobson, M. Dünser, N. Dupont, A. Elliott-Peisert, F. Fallavollita, D. Fasanella, S. Fiorendi, G. Franzoni, J. Fulcher, W. Funk, S. Giani, D. Gigi, A. Gilbert, K. Gill, F. Glege, M. Gruchala, M. Guilbaud, D. Gulhan, J. Hegeman, C. Heidegger, Y. Iiyama, V. Innocente, P. Janot, O. Karacheban, J. Kaspar, J. Kieseler, M. Krammer, C. Lange, P. Lecoq, C. Lourenço, L. Malgeri, M. Mannelli, A. Massironi, F. Meijers, J. A. Merlin, S. Mersi, E. Meschi, F. Moortgat, M. Mulders, J. Ngadiuba, S. Nourbakhsh, S. Orfanelli, L. Orsini, F. Pantaleo, L. Pape, E. Perez, M. Peruzzi, A. Petrilli, G. Petrucciani, A. Pfeiffer, M. Pierini, F. M. Pitters, D. Rabady, A. Racz, M. Rovere, H. Sakulin, C. Schäfer, C. Schwick, M. Selvaggi, A. Sharma, P. Silva, W. Snoeys, P. Sphicas, J. Steggemann, S. Summers, V. R. Tavolaro, D. Treille, A. Tsirou, A. Vartak, M. Verzetti, W. D. Zeuner, L. Caminada, K. Deiters, W. Erdmann, R. Horisberger, Q. Ingram, H. C. Kaestli, D. Kotlinski, U. Langenegger, T. Rohe, S. A. Wiederkehr, M. Backhaus, P. Berger, N. Chernyavskaya, G. Dissertori, M. Dittmar, M. Donegà, C. Dorfer, T. A. Gómez Espinosa, C. Grab, D. Hits, T. Klijnsma, W. Lustermann, R. A. Manzoni, M. Marionneau, M. T. Meinhard, F. Micheli, P. Musella, F. Nessi-Tedaldi, F. Pauss, G. Perrin, L. Perrozzi, S. Pigazzini, M. G. Ratti, M. Reichmann, C. Reissel, T. Reitenspiess, D. Ruini, D. A. Sanz Becerra, M. Schönenberger, L. Shchutska, M. L. Vesterbacka Olsson, R. Wallny, D. H. Zhu, T. K. Aarrestad, C. Amsler, D. Brzhechko, M. F. Canelli, A. De Cosa, R. Del Burgo, S. Donato, B. Kilminster, S. Leontsinis, V. M. Mikuni, I. Neutelings, G. Rauco, P. Robmann, D. Salerno, K. Schweiger, C. Seitz, Y. Takahashi, S. Wertz, A. Zucchetta, T. H. Doan, C. M. Kuo, W. Lin, A. Roy, S. S. Yu, P. Chang, Y. Chao, K. F. Chen, P. H. Chen, W.-S. Hou, Y. y. Li, R.-S. Lu, E. Paganis, A. Psallidas, A. Steen, B. Asavapibhop, C. Asawatangtrakuldee, N. Srimanobhas, N. Suwonjandee, A. Bat, F. Boran, S. Cerci, S. Damarseckin, Z. S. Demiroglu, F. Dolek, C. Dozen, I. Dumanoglu, G. Gokbulut, EmineGurpinar Guler, Y. Guler, I. Hos, C. Isik, E. E. Kangal, O. Kara, A. Kayis Topaksu, U. Kiminsu, M. Oglakci, G. Onengut, K. Ozdemir, S. Ozturk, A. E. Simsek, D. Sunar Cerci, U. G. Tok, S. Turkcapar, I. S. Zorbakir, C. Zorbilmez, B. Isildak, G. Karapinar, M. Yalvac, I. O. Atakisi, E. Gülmez, M. Kaya, O. Kaya, B. Kaynak, Ö. Özçelik, S. Tekten, E. A. Yetkin, A. Cakir, K. Cankocak, Y. Komurcu, S. Sen, S. Ozkorucuklu, B. Grynyov, L. Levchuk, F. Ball, E. Bhal, S. Bologna, J. J. Brooke, D. Burns, E. Clement, D. Cussans, H. Flacher, J. Goldstein, G. P. Heath, H. F. Heath, L. Kreczko, S. Paramesvaran, B. Penning, T. Sakuma, S. Seif El Nasr-Storey, D. Smith, V. J. Smith, J. Taylor, A. Titterton, K. W. Bell, A. Belyaev, C. Brew, R. M. Brown, D. Cieri, D. J. A. Cockerill, J. A. Coughlan, K. Harder, S. Harper, J. Linacre, K. Manolopoulos, D. M. Newbold, E. Olaiya, D. Petyt, T. Reis, T. Schuh, C. H. Shepherd-Themistocleous, A. Thea, I. R. Tomalin, T. Williams, W. J. Womersley, R. Bainbridge, P. Bloch, J. Borg, S. Breeze, O. Buchmuller, A. Bundock, GurpreetSingh CHAHAL, D. Colling, P. Dauncey, G. Davies, M. Della Negra, R. Di Maria, P. Everaerts, G. Hall, G. Iles, T. James, M. Komm, C. Laner, L. Lyons, A.-M. Magnan, S. Malik, A. Martelli, V. Milosevic, J. Nash, V. Palladino, M. Pesaresi, D. M. Raymond, A. Richards, A. Rose, E. Scott, C. Seez, A. Shtipliyski, M. Stoye, T. Strebler, A. Tapper, K. Uchida, T. Virdee, N. Wardle, D. Winterbottom, J. Wright, A. G. Zecchinelli, S. C. Zenz, J. E. Cole, P. R. Hobson, A. Khan, P. Kyberd, C. K. Mackay, A. Morton, I. D. Reid, L. Teodorescu, S. Zahid, K. Call, J. Dittmann, K. Hatakeyama, C. Madrid, B. McMaster, N. Pastika, C. Smith, R. Bartek, A. Dominguez, R. Uniyal, A. Buccilli, S. I. Cooper, C. Henderson, P. Rumerio, C. West, D. Arcaro, T. Bose, Z. Demiragli, D. Gastler, S. Girgis, D. Pinna, C. Richardson, J. Rohlf, D. Sperka, I. Suarez, L. Sulak, D. Zou, G. Benelli, B. Burkle, X. Coubez, D. Cutts, Y. t. Duh, M. Hadley, J. Hakala, U. Heintz, J. M. Hogan, K. H. M. Kwok, E. Laird, G. Landsberg, J. Lee, Z. Mao, M. Narain, S. Sagir, R. Syarif, E. Usai, D. Yu, R. Band, C. Brainerd, R. Breedon, M. Calderon De La Barca Sanchez, M. Chertok, J. Conway, R. Conway, P. T. Cox, R. Erbacher, C. Flores, G. Funk, F. Jensen, W. Ko, O. Kukral, R. Lander, M. Mulhearn, D. Pellett, J. Pilot, M. Shi, D. Taylor, K. Tos, M. Tripathi, Z. Wang, F. Zhang, M. Bachtis, C. Bravo, R. Cousins, A. Dasgupta, A. Florent, J. Hauser, M. Ignatenko, N. Mccoll, W. A. Nash, S. Regnard, D. Saltzberg, C. Schnaible, B. Stone, V. Valuev, K. Burt, R. Clare, J. W. Gary, S. M. A. Ghiasi Shirazi, G. Hanson, G. Karapostoli, E. Kennedy, O. R. Long, M. Olmedo Negrete, M. I. Paneva, W. Si, L. Wang, H. Wei, S. Wimpenny, B. R. Yates, Y. Zhang, J. G. Branson, P. Chang, S. Cittolin, M. Derdzinski, R. Gerosa, D. Gilbert, B. Hashemi, D. Klein, V. Krutelyov, J. Letts, M. Masciovecchio, S. May, S. Padhi, M. Pieri, V. Sharma, M. Tadel, F. Würthwein, A. Yagil, G. Zevi Della Porta, N. Amin, R. Bhandari, C. Campagnari, M. Citron, O. Colegrove, V. Dutta, M. Franco Sevilla, L. Gouskos, J. Incandela, B. Marsh, H. Mei, A. Ovcharova, H. Qu, J. Richman, U. Sarica, D. Stuart, S. Wang, D. Anderson, A. Bornheim, O. Cerri, I. Dutta, J. M. Lawhorn, N. Lu, J. Mao, H. B. Newman, T. Q. Nguyen, J. Pata, M. Spiropulu, J. R. Vlimant, S. Xie, Z. Zhang, R. Y. Zhu, M. B. Andrews, T. Ferguson, T. Mudholkar, M. Paulini, M. Sun, I. Vorobiev, M. Weinberg, J. P. Cumalat, W. T. Ford, A. Johnson, E. MacDonald, T. Mulholland, R. Patel, A. Perloff, K. Stenson, K. A. Ulmer, S. R. Wagner, J. Alexander, J. Chaves, Y. Cheng, J. Chu, A. Datta, A. Frankenthal, K. Mcdermott, J. R. Patterson, D. Quach, A. Rinkevicius, A. Ryd, S. M. Tan, Z. Tao, J. Thom, P. Wittich, M. Zientek, S. Abdullin, M. Albrow, M. Alyari, G. Apollinari, A. Apresyan, A. Apyan, S. Banerjee, L. A. T. Bauerdick, A. Beretvas, J. Berryhill, P. C. Bhat, K. Burkett, J. N. Butler, A. Canepa, G. B. Cerati, H. W. K. Cheung, F. Chlebana, M. Cremonesi, J. Duarte, V. D. Elvira, J. Freeman, Z. Gecse, E. Gottschalk, L. Gray, D. Green, S. Grünendahl, O. Gutsche, AllisonReinsvold Hall, J. Hanlon, R. M. Harris, S. Hasegawa, R. Heller, J. Hirschauer, B. Jayatilaka, S. Jindariani, M. Johnson, U. Joshi, B. Klima, M. J. Kortelainen, B. Kreis, S. Lammel, J. Lewis, D. Lincoln, R. Lipton, M. Liu, T. Liu, J. Lykken, K. Maeshima, J. M. Marraffino, D. Mason, P. McBride, P. Merkel, S. Mrenna, S. Nahn, V. O’Dell, V. Papadimitriou, K. Pedro, C. Pena, G. Rakness, F. Ravera, L. Ristori, B. Schneider, E. Sexton-Kennedy, N. Smith, A. Soha, W. J. Spalding, L. Spiegel, S. Stoynev, J. Strait, N. Strobbe, L. Taylor, S. Tkaczyk, N. V. Tran, L. Uplegger, E. W. Vaandering, C. Vernieri, M. Verzocchi, R. Vidal, M. Wang, H. A. Weber, D. Acosta, P. Avery, D. Bourilkov, A. Brinkerhoff, L. Cadamuro, A. Carnes, V. Cherepanov, D. Curry, F. Errico, R. D. Field, S. V. Gleyzer, B. M. Joshi, M. Kim, J. Konigsberg, A. Korytov, K. H. Lo, P. Ma, K. Matchev, N. Menendez, G. Mitselmakher, D. Rosenzweig, K. Shi, J. Wang, S. Wang, X. Zuo, Y. R. Joshi, T. Adams, A. Askew, S. Hagopian, V. Hagopian, K. F. Johnson, R. Khurana, T. Kolberg, G. Martinez, T. Perry, H. Prosper, C. Schiber, R. Yohay, J. Zhang, M. M. Baarmand, V. Bhopatkar, M. Hohlmann, D. Noonan, M. Rahmani, M. Saunders, F. Yumiceva, M. R. Adams, L. Apanasevich, D. Berry, R. R. Betts, R. Cavanaugh, X. Chen, S. Dittmer, O. Evdokimov, C. E. Gerber, D. A. Hangal, D. J. Hofman, K. Jung, C. Mills, T. Roy, M. B. Tonjes, N. Varelas, H. Wang, X. Wang, Z. Wu, M. Alhusseini, B. Bilki, W. Clarida, K. Dilsiz, S. Durgut, R. P. Gandrajula, M. Haytmyradov, V. Khristenko, O. K. Köseyan, J.-P. Merlo, A. Mestvirishvili, A. Moeller, J. Nachtman, H. Ogul, Y. Onel, F. Ozok, A. Penzo, C. Snyder, E. Tiras, J. Wetzel, B. Blumenfeld, A. Cocoros, N. Eminizer, D. Fehling, L. Feng, A. V. Gritsan, W. T. Hung, P. Maksimovic, J. Roskes, M. Swartz, M. Xiao, C. Baldenegro Barrera, P. Baringer, A. Bean, S. Boren, J. Bowen, A. Bylinkin, T. Isidori, S. Khalil, J. King, G. Krintiras, A. Kropivnitskaya, C. Lindsey, D. Majumder, W. Mcbrayer, N. Minafra, M. Murray, C. Rogan, C. Royon, S. Sanders, E. Schmitz, J. D. Tapia Takaki, Q. Wang, J. Williams, G. Wilson, S. Duric, A. Ivanov, K. Kaadze, D. Kim, Y. Maravin, D. R. Mendis, T. Mitchell, A. Modak, A. Mohammadi, F. Rebassoo, D. Wright, A. Baden, O. Baron, A. Belloni, S. C. Eno, Y. Feng, N. J. Hadley, S. Jabeen, G. Y. Jeng, R. G. Kellogg, J. Kunkle, A. C. Mignerey, S. Nabili, F. Ricci-Tam, M. Seidel, Y. H. Shin, A. Skuja, S. C. Tonwar, K. Wong, D. Abercrombie, B. Allen, A. Baty, R. Bi, S. Brandt, W. Busza, I. A. Cali, M. D’Alfonso, G. Gomez Ceballos, M. Goncharov, P. Harris, D. Hsu, M. Hu, M. Klute, D. Kovalskyi, Y.-J. Lee, P. D. Luckey, B. Maier, A. C. Marini, C. Mcginn, C. Mironov, S. Narayanan, X. Niu, C. Paus, D. Rankin, C. Roland, G. Roland, Z. Shi, G. S. F. Stephans, K. Sumorok, K. Tatar, D. Velicanu, J. Wang, T. W. Wang, B. Wyslouch, A. C. Benvenuti, R. M. Chatterjee, A. Evans, S. Guts, P. Hansen, J. Hiltbrand, Sh. Jain, Y. Kubota, Z. Lesko, J. Mans, R. Rusack, M. A. Wadud, J. G. Acosta, S. Oliveros, K. Bloom, D. R. Claes, C. Fangmeier, L. Finco, F. Golf, R. Gonzalez Suarez, R. Kamalieddin, I. Kravchenko, J. E. Siado, G. R. Snow, B. Stieger, G. Agarwal, C. Harrington, I. Iashvili, A. Kharchilava, C. McLean, D. Nguyen, A. Parker, J. Pekkanen, S. Rappoccio, B. Roozbahani, G. Alverson, E. Barberis, C. Freer, Y. Haddad, A. Hortiangtham, G. Madigan, D. M. Morse, T. Orimoto, L. Skinnari, A. Tishelman-Charny, T. Wamorkar, B. Wang, A. Wisecarver, D. Wood, S. Bhattacharya, J. Bueghly, T. Gunter, K. A. Hahn, N. Odell, M. H. Schmitt, K. Sung, M. Trovato, M. Velasco, R. Bucci, N. Dev, R. Goldouzian, M. Hildreth, K. Hurtado Anampa, C. Jessop, D. J. Karmgard, K. Lannon, W. Li, N. Loukas, N. Marinelli, I. Mcalister, F. Meng, C. Mueller, Y. Musienko, M. Planer, R. Ruchti, P. Siddireddy, G. Smith, S. Taroni, M. Wayne, A. Wightman, M. Wolf, A. Woodard, J. Alimena, B. Bylsma, L. S. Durkin, S. Flowers, B. Francis, C. Hill, W. Ji, A. Lefeld, T. Y. Ling, B. L. Winer, S. Cooperstein, G. Dezoort, P. Elmer, J. Hardenbrook, N. Haubrich, S. Higginbotham, A. Kalogeropoulos, S. Kwan, D. Lange, M. T. Lucchini, J. Luo, D. Marlow, K. Mei, I. Ojalvo, J. Olsen, C. Palmer, P. Piroué, J. Salfeld-Nebgen, D. Stickland, C. Tully, Z. Wang, S. Malik, S. Norberg, A. Barker, V. E. Barnes, S. Das, L. Gutay, M. Jones, A. W. Jung, A. Khatiwada, B. Mahakud, D. H. Miller, G. Negro, N. Neumeister, C. C. Peng, S. Piperov, H. Qiu, J. F. Schulte, J. Sun, F. Wang, R. Xiao, W. Xie, T. Cheng, J. Dolen, N. Parashar, K. M. Ecklund, S. Freed, F. J. M. Geurts, M. Kilpatrick, Arun Kumar, W. Li, B. P. Padley, R. Redjimi, J. Roberts, J. Rorie, W. Shi, A. G. Stahl Leiton, Z. Tu, A. Zhang, A. Bodek, P. de Barbaro, R. Demina, J. L. Dulemba, C. Fallon, T. Ferbel, M. Galanti, A. Garcia-Bellido, J. Han, O. Hindrichs, A. Khukhunaishvili, E. Ranken, P. Tan, R. Taus, B. Chiarito, J. P. Chou, A. Gandrakota, Y. Gershtein, E. Halkiadakis, A. Hart, M. Heindl, E. Hughes, S. Kaplan, S. Kyriacou, I. Laflotte, A. Lath, R. Montalvo, K. Nash, M. Osherson, H. Saka, S. Salur, S. Schnetzer, D. Sheffield, S. Somalwar, R. Stone, S. Thomas, P. Thomassen, H. Acharya, A. G. Delannoy, G. Riley, S. Spanier, O. Bouhali, A. Celik, M. Dalchenko, M. De Mattia, A. Delgado, S. Dildick, R. Eusebi, J. Gilmore, T. Huang, T. Kamon, S. Luo, D. Marley, R. Mueller, D. Overton, L. Perniè, D. Rathjens, A. Safonov, N. Akchurin, J. Damgov, F. De Guio, S. Kunori, K. Lamichhane, S. W. Lee, T. Mengke, S. Muthumuni, T. Peltola, S. Undleeb, I. Volobouev, Z. Wang, A. Whitbeck, S. Greene, A. Gurrola, R. Janjam, W. Johns, C. Maguire, A. Melo, H. Ni, K. Padeken, F. Romeo, P. Sheldon, S. Tuo, J. Velkovska, M. Verweij, M. W. Arenton, P. Barria, B. Cox, G. Cummings, R. Hirosky, M. Joyce, A. Ledovskoy, C. Neu, B. Tannenwald, Y. Wang, E. Wolfe, F. Xia, R. Harr, P. E. Karchin, N. Poudyal, J. Sturdy, P. Thapa, S. Zaleski, J. Buchanan, C. Caillol, D. Carlsmith, S. Dasu, I. De Bruyn, L. Dodd, F. Fiori, C. Galloni, B. Gomber, H. He, M. Herndon, A. Hervé, U. Hussain, P. Klabbers, A. Lanaro, A. Loeliger, K. Long, R. Loveless, J. Madhusudanan Sreekala, T. Ruggles, A. Savin, V. Sharma, W. H. Smith, D. Teague, S. Trembath-reichert, N. Woods

**Affiliations:** 1 Yerevan Physics Institute, Yerevan, Armenia; 2 Institut für Hochenergiephysik, Wien, Austria; 3 Institute for Nuclear Problems, Minsk, Belarus; 4 Universiteit Antwerpen, Antwerpen, Belgium; 5 Vrije Universiteit Brussel, Brussel, Belgium; 6 Université Libre de Bruxelles, Bruxelles, Belgium; 7 Ghent University, Ghent, Belgium; 8 Université Catholique de Louvain, Louvain-la-Neuve, Belgium; 9 Centro Brasileiro de Pesquisas Fisicas, Rio de Janeiro, Brazil; 10 Universidade do Estado do Rio de Janeiro, Rio de Janeiro, Brazil; 11 Universidade Estadual Paulista, Universidade Federal do ABC, São Paulo, Brazil; 12 Institute for Nuclear Research and Nuclear Energy, Bulgarian Academy of Sciences, Sofia, Bulgaria; 13 University of Sofia, Sofia, Bulgaria; 14 Beihang University, Beijing, China; 15 Institute of High Energy Physics, Beijing, China; 16 State Key Laboratory of Nuclear Physics and Technology, Peking University, Beijing, China; 17 Tsinghua University, Beijing, China; 18 Universidad de Los Andes, Bogota, Colombia; 19 Universidad de Antioquia, Medellin, Colombia; 20 University of Split, Faculty of Electrical Engineering, Mechanical Engineering and Naval Architecture, Split, Croatia; 21 University of Split, Faculty of Science, Split, Croatia; 22 Institute Rudjer Boskovic, Zagreb, Croatia; 23 University of Cyprus, Nicosia, Cyprus; 24 Charles University, Prague, Czech Republic; 25 Escuela Politecnica Nacional, Quito, Ecuador; 26 Universidad San Francisco de Quito, Quito, Ecuador; 27 Academy of Scientific Research and Technology of the Arab Republic of Egypt, Egyptian Network of High Energy Physics, Cairo, Egypt; 28 National Institute of Chemical Physics and Biophysics, Tallinn, Estonia; 29 Department of Physics, University of Helsinki, Helsinki, Finland; 30 Helsinki Institute of Physics, Helsinki, Finland; 31 Lappeenranta University of Technology, Lappeenranta, Finland; 32 IRFU, CEA, Université Paris-Saclay, Gif-sur-Yvette, France; 33 Laboratoire Leprince-Ringuet, CNRS/IN2P3, Ecole Polytechnique, Institut Polytechnique de Paris, Palaiseau, France; 34 Université de Strasbourg, CNRS, IPHC UMR 7178, Strasbourg, France; 35 Centre de Calcul de l’Institut National de Physique Nucleaire et de Physique des Particules, CNRS/IN2P3, Villeurbanne, France; 36 Université de Lyon, Université Claude Bernard Lyon 1, CNRS-IN2P3, Institut de Physique Nucléaire de Lyon, Villeurbanne, France; 37 Georgian Technical University, Tbilisi, Georgia; 38 Tbilisi State University, Tbilisi, Georgia; 39 RWTH Aachen University, I. Physikalisches Institut, Aachen, Germany; 40 RWTH Aachen University, III. Physikalisches Institut A, Aachen, Germany; 41 RWTH Aachen University, III. Physikalisches Institut B, Aachen, Germany; 42 Deutsches Elektronen-Synchrotron, Hamburg, Germany; 43 University of Hamburg, Hamburg, Germany; 44 Karlsruher Institut fuer Technologie, Karlsruhe, Germany; 45 Institute of Nuclear and Particle Physics (INPP), NCSR Demokritos, Aghia Paraskevi, Greece; 46 National and Kapodistrian University of Athens, Athens, Greece; 47 National Technical University of Athens, Athens, Greece; 48 University of Ioánnina, Ioánnina, Greece; 49 MTA-ELTE Lendület CMS Particle and Nuclear Physics Group, Eötvös Loránd University, Budapest, Hungary; 50 Wigner Research Centre for Physics, Budapest, Hungary; 51 Institute of Nuclear Research ATOMKI, Debrecen, Hungary; 52 Institute of Physics, University of Debrecen, Debrecen, Hungary; 53 Eszterhazy Karoly University, Karoly Robert Campus, Gyongyos, Hungary; 54 Indian Institute of Science (IISc), Bangalore, India; 55 National Institute of Science Education and Research, HBNI, Bhubaneswar, India; 56 Panjab University, Chandigarh, India; 57 University of Delhi, Delhi, India; 58 Saha Institute of Nuclear Physics, HBNI, Kolkata, India; 59 Indian Institute of Technology Madras, Madras, India; 60 Bhabha Atomic Research Centre, Mumbai, India; 61 Tata Institute of Fundamental Research-A, Mumbai, India; 62 Tata Institute of Fundamental Research-B, Mumbai, India; 63 Indian Institute of Science Education and Research (IISER), Pune, India; 64 Institute for Research in Fundamental Sciences (IPM), Tehran, Iran; 65 University College Dublin, Dublin, Ireland; 66 INFN Sezione di Bari, Università di Bari, Politecnico di Bari, Bari, Italy; 67 INFN Sezione di Bologna, Università di Bologna, Bologna, Italy; 68 INFN Sezione di Catania, Università di Catania, Catania, Italy; 69 INFN Sezione di Firenze, Università di Firenze, Firenze, Italy; 70 INFN Laboratori Nazionali di Frascati, Frascati, Italy; 71 INFN Sezione di Genova, Università di Genova, Genova, Italy; 72 INFN Sezione di Milano-Bicocca, Università di Milano-Bicocca, Milan, Italy; 73 INFN Sezione di Napoli, Università di Napoli ’Federico II’ , Napoli, Italy, Università della Basilicata, Potenza, Italy, Università G. Marconi, Rome, Italy; 74 INFN Sezione di Padova, Università di Padova, Padua, Italy, Università di Trento, Trento, Italy; 75 INFN Sezione di Pavia, Università di Pavia, Pavia, Italy; 76 INFN Sezione di Perugia, Università di Perugia, Perugia, Italy; 77 INFN Sezione di Pisa, Università di Pisa, Scuola Normale Superiore di Pisa, Pisa, Italy; 78 INFN Sezione di Roma, Sapienza Università di Roma, Rome, Italy; 79 INFN Sezione di Torino, Università di Torino, Torino, Italy, Università del Piemonte Orientale, Novara, Italy; 80 INFN Sezione di Trieste, Università di Trieste, Trieste, Italy; 81 Kyungpook National University, Daegu, Korea; 82 Chonnam National University, Institute for Universe and Elementary Particles, Kwangju, Korea; 83 Hanyang University, Seoul, Korea; 84 Korea University, Seoul, Korea; 85 Department of Physics, Kyung Hee University, Seoul, South Korea; 86 Sejong University, Seoul, Korea; 87 Seoul National University, Seoul, Korea; 88 University of Seoul, Seoul, Korea; 89 Sungkyunkwan University, Suwon, Korea; 90 Riga Technical University, Riga, Latvia; 91 Vilnius University, Vilnius, Lithuania; 92 National Centre for Particle Physics, Universiti Malaya, Kuala Lumpur, Malaysia; 93 Universidad de Sonora (UNISON), Hermosillo, Mexico; 94 Centro de Investigacion y de Estudios Avanzados del IPN, Mexico City, Mexico; 95 Universidad Iberoamericana, Mexico City, Mexico; 96 Benemerita Universidad Autonoma de Puebla, Puebla, Mexico; 97 Universidad Autónoma de San Luis Potosí, San Luis Potosí, Mexico; 98 University of Montenegro, Podgorica, Montenegro; 99 University of Auckland, Auckland, New Zealand; 100 University of Canterbury, Christchurch, New Zealand; 101 National Centre for Physics, Quaid-I-Azam University, Islamabad, Pakistan; 102 AGH University of Science and Technology Faculty of Computer Science, Electronics and Telecommunications, Krakow, Poland; 103 National Centre for Nuclear Research, Swierk, Poland; 104 Institute of Experimental Physics, Faculty of Physics, University of Warsaw, Warsaw, Poland; 105 Laboratório de Instrumentação e Física Experimental de Partículas, Lisboa, Portugal; 106 Joint Institute for Nuclear Research, Dubna, Russia; 107 Petersburg Nuclear Physics Institute, Gatchina (St. Petersburg), Russia; 108 Institute for Nuclear Research, Moscow, Russia; 109 Institute for Theoretical and Experimental Physics named by A.I. Alikhanov of NRC ‘Kurchatov Institute’, Moscow, Russia; 110 Moscow Institute of Physics and Technology, Moscow, Russia; 111 National Research Nuclear University ’Moscow Engineering Physics Institute’ (MEPhI), Moscow, Russia; 112 P.N. Lebedev Physical Institute, Moscow, Russia; 113 Skobeltsyn Institute of Nuclear Physics, Lomonosov Moscow State University, Moscow, Russia; 114 Novosibirsk State University (NSU), Novosibirsk, Russia; 115 Institute for High Energy Physics of National Research Centre ‘Kurchatov Institute’, Protvino, Russia; 116 National Research Tomsk Polytechnic University, Tomsk, Russia; 117 Tomsk State University, Tomsk, Russia; 118 University of Belgrade: Faculty of Physics and VINCA Institute of Nuclear Sciences, Belgrade, Serbia; 119 Centro de Investigaciones Energéticas Medioambientales y Tecnológicas (CIEMAT), Madrid, Spain; 120 Universidad Autónoma de Madrid, Madrid, Spain; 121 Universidad de Oviedo, Instituto Universitario de Ciencias y Tecnologías Espaciales de Asturias (ICTEA), Oviedo, Spain; 122 Instituto de Física de Cantabria (IFCA), CSIC-Universidad de Cantabria, Santander, Spain; 123 University of Colombo, Colombo, Sri Lanka; 124 Department of Physics, University of Ruhuna, Matara, Sri Lanka; 125 CERN, European Organization for Nuclear Research, Geneva, Switzerland; 126 Paul Scherrer Institut, Villigen, Switzerland; 127 ETH Zurich-Institute for Particle Physics and Astrophysics (IPA), Zurich, Switzerland; 128 Universität Zürich, Zurich, Switzerland; 129 National Central University, Chung-Li, Taiwan; 130 National Taiwan University (NTU), Taipei, Taiwan; 131 Chulalongkorn University, Faculty of Science, Department of Physics, Bangkok, Thailand; 132 ukurova University, Physics Department, Science and Art Faculty, Adana, Turkey; 133 Middle East Technical University, Physics Department, Ankara, Turkey; 134 Bogazici University, Istanbul, Turkey; 135 Istanbul Technical University, Istanbul, Turkey; 136 Istanbul University, Istanbul, Turkey; 137 Institute for Scintillation Materials of National Academy of Science of Ukraine, Kharkov, Ukraine; 138 National Scientific Center, Kharkov Institute of Physics and Technology, Kharkov, Ukraine; 139 University of Bristol, Bristol, UK; 140 Rutherford Appleton Laboratory, Didcot, UK; 141 Imperial College, London, UK; 142 Brunel University, Uxbridge, UK; 143 Baylor University, Waco, USA; 144 Catholic University of America, Washington DC, USA; 145 The University of Alabama, Tuscaloosa, USA; 146 Boston University, Boston, USA; 147 Brown University, Providence, USA; 148 University of California, Davis, Davis USA; 149 University of California, Los Angeles, USA; 150 University of California, Riverside, Riverside, USA; 151 University of California, San Diego, La Jolla, USA; 152 University of California, Santa Barbara-Department of Physics, Santa Barbara, USA; 153 California Institute of Technology, Pasadena, USA; 154 Carnegie Mellon University, Pittsburgh, USA; 155 University of Colorado Boulder, Boulder, USA; 156 Cornell University, Ithaca, USA; 157 Fermi National Accelerator Laboratory, Batavia, USA; 158 University of Florida, Gainesville, USA; 159 Florida International University, Miami, USA; 160 Florida State University, Tallahassee, USA; 161 Florida Institute of Technology, Melbourne, USA; 162 University of Illinois at Chicago (UIC), Chicago, USA; 163 The University of Iowa, Iowa City, USA; 164 Johns Hopkins University, Baltimore, USA; 165 The University of Kansas, Lawrence, USA; 166 Kansas State University, Manhattan, USA; 167 Lawrence Livermore National Laboratory, Livermore, USA; 168 University of Maryland, College Park, USA; 169 Massachusetts Institute of Technology, Cambridge, USA; 170 University of Minnesota, Minneapolis, USA; 171 University of Mississippi, Oxford, USA; 172 University of Nebraska-Lincoln, Lincoln, USA; 173 State University of New York at Buffalo, Buffalo, USA; 174 Northeastern University, Boston, USA; 175 Northwestern University, Evanston, USA; 176 University of Notre Dame, Notre Dame, USA; 177 The Ohio State University, Columbus, USA; 178 Princeton University, Princeton, USA; 179 University of Puerto Rico, Mayaguez, USA; 180 Purdue University, West Lafayette, USA; 181 Purdue University Northwest, Hammond, USA; 182 Rice University, Houston, USA; 183 University of Rochester, Rochester, USA; 184 Rutgers, The State University of New Jersey, Piscataway, USA; 185 University of Tennessee, Knoxville, USA; 186 Texas A & M University, College Station, USA; 187 Texas Tech University, Lubbock, USA; 188 Vanderbilt University, Nashville, USA; 189 University of Virginia, Charlottesville, USA; 190 Wayne State University, Detroit, USA; 191 University of Wisconsin-Madison, Madison, WI USA

## Abstract

A search is presented for ${\uptau }_{}^{}$ slepton pairs produced in proton–proton collisions at a center-of-mass energy of 13$\,\text {TeV}$. The search is carried out in events containing two ${\uptau }_{}^{}$ leptons in the final state, on the assumption that each ${\uptau }_{}^{}$ slepton decays primarily to a ${\uptau }_{}^{}$ lepton and a neutralino. Events are considered in which each ${\uptau }_{}^{}$ lepton decays to one or more hadrons and a neutrino, or in which one of the ${\uptau }_{}^{}$ leptons decays instead to an electron or a muon and two neutrinos. The data, collected with the CMS detector in 2016 and 2017, correspond to an integrated luminosity of 77.2$\,\text {fb}^{-1}$. The observed data are consistent with the standard model background expectation. The results are used to set 95% confidence level upper limits on the cross section for ${\uptau }_{}^{}$ slepton pair production in various models for ${\uptau }_{}^{}$ slepton masses between 90 and 200$\,\text {GeV}$ and neutralino masses of 1, 10, and 20$\,\text {GeV}$. In the case of purely left-handed ${\uptau }_{}^{}$ slepton production and decay to a ${\uptau }_{}^{}$ lepton and a neutralino with a mass of 1$\,\text {GeV}$, the strongest limit is obtained for a ${\uptau }_{}^{}$ slepton mass of 125$\,\text {GeV}$ at a factor of 1.14 larger than the theoretical cross section.

## Introduction

Supersymmetry (SUSY) [1–8] is a possible extension of the standard model (SM) of particle physics, characterized by the presence of superpartners for SM particles. The superpartners have the same quantum numbers as their SM counterparts, except for the spin, which differs by half a unit. One appealing feature of SUSY is that the cancellation of quadratic divergences in quantum corrections to the Higgs boson mass from SM particles and their superpartners could resolve the fine tuning problem [9–12]. Another feature is that the lightest supersymmetric particle (LSP) is stable in SUSY models with *R*-parity conservation [13], and could be a dark matter (DM) candidate [14–16].

The hypothetical superpartner of the ${\uptau }_{}^{}$ lepton, the ${\uptau }_{}^{}$ slepton ($\widetilde{\uptau }_{}^{}$), is the focus of the search reported in this paper. Supersymmetric models where a light $\widetilde{\uptau }_{}^{}$ is the next-to-lightest supersymmetric particle are well motivated in early universe $\widetilde{\uptau }_{}^{}$-neutralino coannihilation models that can accommodate the observed DM relic density [17–22]. The existence of a light $\widetilde{\uptau }_{}^{}$ would enhance the rate of production of final states with ${\uptau }_{}^{}$ leptons in collider experiments [23, 24].

**Fig. 1:**
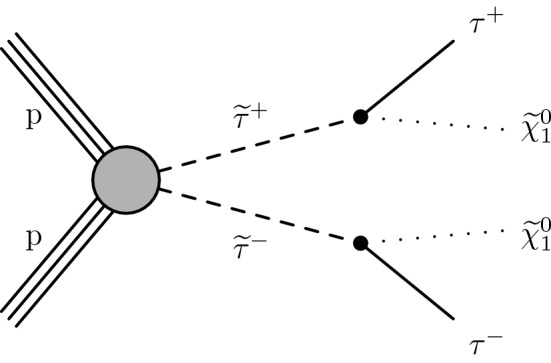
Diagram for direct $\widetilde{\uptau }_{}^{}$ pair production, followed by decay of each $\widetilde{\uptau }_{}^{}$ to a ${\uptau }_{}^{}$ lepton and a ${\widetilde{\upchi }}_{1}^{0}$

In this analysis, we study the simplified model [25–27] of direct $\widetilde{\uptau }_{}^{}$ pair production shown in Fig. 1. We assume that the $\widetilde{\uptau }_{}^{}$ decays to a ${\uptau }_{}^{}$ lepton and ${\widetilde{\upchi }}_{1}^{0}$, the lightest neutralino, which is the LSP in this model. The search is challenging because of the extremely small production cross section expected for this signal, as well as the large backgrounds. The most sensitive previous searches for direct $\widetilde{\uptau }_{}^{}$ pair production were performed at the CERN LEP collider [28–31], excluding $\widetilde{\uptau }_{}^{}$ masses at 95% confidence level ($\text {CL}$) up to $\approx$90$\,\text {GeV}$ for neutralino masses up to 80$\,\text {GeV}$ in some models. At the LHC, the ATLAS [32, 33] and CMS [34] Collaborations have also performed searches for direct $\widetilde{\uptau }_{}^{}$ pair production using 8$\,\text {TeV}$ data, and the CMS Collaboration has reported a search for direct $\widetilde{\uptau }_{}^{}$ pair production in an initial sample of 35.9$\,\text {fb}^{-1}$ at 13$\,\text {TeV}$ collected in 2016 [35]. This paper presents a significant improvement in search sensitivity, which was limited by the small signal production rates, through the incorporation of improved analysis techniques and the inclusion of the data collected in 2017. The data used correspond to a total integrated luminosity of 77.2$\,\text {fb}^{-1}$.

Events with two ${\uptau }_{}^{}$ leptons are used. We consider both hadronic and leptonic decay modes of the ${\uptau }_{}^{}$ lepton, in which it decays to one or more hadrons and a neutrino, or to an electron or muon and two neutrinos, respectively. Independent analyses are carried out in the final states with two hadronically decaying ${\uptau }_{}^{}$ leptons (${{\uptau }_{}^{}} _\mathrm {h} {{\uptau }_{}^{}} _\mathrm {h}$) and with one ${{\uptau }_{}^{}} _\mathrm {h}$ and an electron or a muon ($\ell {{\uptau }_{}^{}} _\mathrm {h}$, where $\ell = {{\hbox {e}}_{}^{}}$ or ${\upmu }_{}^{}$). The presence of missing transverse momentum, which can originate from stable neutralinos as well as neutrinos from ${\uptau }_{}^{}$ lepton decays, provides an important source of discriminating power between signal and background.

We have introduced several improvements with respect to the analysis presented in Ref. [35] that are applied to both 2016 and 2017 data. We make use of dedicated machine learning techniques to enhance the search sensitivity. These include the incorporation of an improved ${{\uptau }_{}^{}} _\mathrm {h}$ selection method that makes use of a deep neural network (DNN) for the ${{\uptau }_{}^{}} _\mathrm {h} {{\uptau }_{}^{}} _\mathrm {h}$ analysis, and of a boosted decision tree (BDT) for event selection in the $\ell {{\uptau }_{}^{}} _\mathrm {h}$ analyses. Improvements have also been made to the background-estimation techniques and to the search region (SR) definitions. The incorporation of these enhancements is expected to improve the search sensitivity by up to 50%, where the figure of merit considered is the 95% $\text {CL}$ upper limit on the cross section for $\widetilde{\uptau }_{}^{}$ pair production obtained with the data collected in 2016. The improvement is less significant than expected, since it is found that the estimated signal acceptance is reduced when the fast detector simulation that was previously used to model signal events is replaced in this search with the more realistic, full Geant4-based detector simulation [36]. Differences in the signal acceptance for the fast and more accurate full detector simulations are mainly caused by differences in the reconstructed ${{\uptau }_{}^{}} _\mathrm {h}$ visible transverse momentum ($p_{\mathrm {T}}$), which is found to have larger values in the case of the fast simulation.

We consider the superpartners of both left- and right-handed ${\uptau }_{}^{}$ leptons, ${\widetilde{\uptau }}_{\mathrm {L}}$ and ${\widetilde{\uptau }}_{\mathrm {R}}$. The cross section for ${\widetilde{\uptau }}_{\mathrm {L}}$ pair production is expected to be about a factor of three larger than for ${\widetilde{\uptau }}_{\mathrm {R}}$ pairs [37]. The experimental acceptance is also expected to be different for left- and right-handed assignments because of the differences in the polarization of the ${\uptau }_{}^{}$ leptons produced in ${\widetilde{\uptau }}_{\mathrm {L}}$ and ${\widetilde{\uptau }}_{\mathrm {R}}$ decays. The decay products of hadronically and leptonically decaying ${\uptau }_{}^{}$ leptons originating from ${\widetilde{\uptau }}_{\mathrm {R}}$ decays are predicted to have larger and smaller $p_{\mathrm {T}}$, respectively, than those originating from ${\widetilde{\uptau }}_{\mathrm {L}}$ decays. Two simplified models are studied for direct $\widetilde{\uptau }_{}^{}$ pair production. One model involves production of only ${\widetilde{\uptau }}_{\mathrm {L}}$ pairs and the other is for the degenerate case in which both ${\widetilde{\uptau }}_{\mathrm {L}}$ and ${\widetilde{\uptau }}_{\mathrm {R}}$ pairs are produced. No mixing is introduced between left- and right-handed states. We study models with $\widetilde{\uptau }_{}^{}$ masses ranging from 90 to 200$\,\text {GeV}$. The LEP limits [28–31] place strong constraints on the allowed values of the $\widetilde{\uptau }_{}^{}$ mass below this range, while the search sensitivity for $\widetilde{\uptau }_{}^{}$ masses above this range is low as a result of the decrease in production cross section with increased mass. We also consider different assumptions for the ${\widetilde{\upchi }}_{1}^{0}$ mass, namely 1, 10, and 20$\,\text {GeV}$. The search sensitivity decreases when the mass difference between the $\widetilde{\uptau }_{}^{}$ and ${\widetilde{\upchi }}_{1}^{0}$ becomes small, since the visible decay products in such cases have lower momentum, resulting in a loss of experimental acceptance for such signals.

## The CMS detector

The central feature of the CMS apparatus is a superconducting solenoid of 6$\text { m}$ internal diameter, providing a magnetic field of 3.8$\text { T}$. A silicon pixel and strip tracker, a lead tungstate crystal electromagnetic calorimeter (ECAL), and a brass and scintillator hadron calorimeter, each composed of a barrel and two endcap sections, reside within the solenoid volume. Forward calorimeters extend the pseudorapidity ($\eta$) coverage provided by the barrel and endcap detectors. Muons are detected in gas-ionization chambers embedded in the steel flux-return yoke outside the solenoid. Events of interest are selected using a two-tiered trigger system [38]. The first level, composed of custom hardware processors, uses information from the calorimeters and muon detectors to select events at a rate of around 100$\text { kHz}$ within a time interval of less than 4$\,\mu \text {s}$. The second level, known as the high-level trigger, consists of a farm of processors running a version of the full event reconstruction software optimized for fast processing, which reduces the event rate to about 1$\text { kHz}$ before data storage. A more detailed description of the CMS detector, together with definitions of the coordinate system and kinematic variables, can be found in Ref. [39].

## Event reconstruction and simulation

The event reconstruction uses a particle-flow (PF) algorithm [40] that combines information from the tracker, calorimeter, and muon systems to identify charged and neutral hadrons, photons, electrons, and muons in an event. The missing transverse momentum vector, ${\vec p}_{\mathrm {T}}^{\text {miss}}$, is computed as the negative of the vector sum of the $p_{\mathrm {T}}$ of all PF candidates reconstructed in an event, and its magnitude $p_{\mathrm {T}} ^\text {miss}$ is used in the search as a discriminator between signal and SM background. Events selected for the search are required to pass filters [41] designed to remove detector- and beam-related backgrounds, and must have at least one reconstructed vertex. Usually, more than one such vertex is reconstructed because of pileup, i.e., multiple proton–proton (${{\hbox {p}}_{}^{}} {{\hbox {p}}_{}^{}}$) collisions within the same or neighboring bunch crossings. The mean number of interactions per bunch crossing was 27 in 2016, and increased to 37 in 2017, assuming a total inelastic ${{\hbox {p}}_{}^{}} {{\hbox {p}}_{}^{}}$ cross section of 80$\text { mb}$. The reconstructed vertex with the largest value in summed object $p_{\mathrm {T}} ^2$ is selected to be the primary ${{\hbox {p}}_{}^{}} {{\hbox {p}}_{}^{}}$ interaction vertex (PV). These objects are defined by tracks associated with a given vertex that are clustered using a jet finding algorithm [42, 43], and a more restricted form of the vector missing transverse momentum that is calculated from these track-based jets.

Charged particles that originate from the PV, photons, and neutral hadrons are clustered into jets using the anti-$k_{\mathrm {T}}$ algorithm [42] with a distance parameter of 0.4, as implemented in the FastJet package [43]. The jet energies are corrected to account for the contribution from pileup interactions and to compensate for variations in the detector response [43, 44]. To mitigate issues related to noise in the ECAL endcaps that led to significantly worse modeling of the $p_{\mathrm {T}} ^\text {miss}$ distribution, particularly for events with large values of $p_{\mathrm {T}} ^\text {miss}$ in 2017 data, PF candidates that are clustered in jets in $2.65< |\eta | < 3.14$ with uncorrected $p_{\mathrm {T}} <50\,\text {GeV}$ are not used in the calculation of ${\vec p}_{\mathrm {T}}^{\text {miss}}$ in 2017 data and simulation. Disagreements between the $p_{\mathrm {T}} ^\text {miss}$ distributions in data and simulation ranging up to >100% for $50< p_{\mathrm {T}} ^\text {miss} < 170\,\text {GeV}$ in DY+jets events, in which large values of $p_{\mathrm {T}} ^\text {miss}$ arise mainly from mismeasurements, are reduced by this modification of the ${\vec p}_{\mathrm {T}}^{\text {miss}}$ calculation. The modified $p_{\mathrm {T}} ^\text {miss}$ distributions in simulated events and data agree within uncertainties.

Jets in the search are required to have their axes within the tracker volume of $|\eta | < 2.4$. For the ${{\uptau }_{}^{}} _\mathrm {h} {{\uptau }_{}^{}} _\mathrm {h}$ analysis, we use jets with $p_{\mathrm {T}} >30\,\text {GeV}$, while for the $\ell {{\uptau }_{}^{}} _\mathrm {h}$ analyses, we veto events containing jets with $p_{\mathrm {T}} >20$ $\,\text {GeV}$ to provide efficient background rejection. Jets are required to be separated in $\eta$ and azimuthal angle ($\phi$) by $\varDelta R \equiv \sqrt{\smash [b]{(\varDelta \eta )^2 + (\varDelta \phi )^2} } > 0.4$ from electron, muon, or ${{\uptau }_{}^{}} _\mathrm {h}$ candidates in order to minimize double counting of objects. Jets originating from the hadronization of ${\hbox {b}}_{}^{}$ quarks are “tagged” in the ${{\uptau }_{}^{}} _\mathrm {h} {{\uptau }_{}^{}} _\mathrm {h}$ analysis through the DNN-based combined secondary vertex algorithm (DeepCSV) [45] to reject events with ${\hbox {b}}_{}^{}$ quark jets that are likely to originate from backgrounds with top quarks. The efficiency for tagging ${\hbox {b}}_{}^{}$ quarks originating from top quark decays is about 84%, while the misidentification rates for jets from charm quarks, and from light quarks or gluons, are about 41 and 11%, respectively. In the $\ell {{\uptau }_{}^{}} _\mathrm {h}$ analyses, the CSVv2 tagger [45] is used to identify ${\hbox {b}}_{}^{}$ quark jets for the selection of background-enriched control regions (CRs). The working point that is used corresponds to an efficiency of 63% and misidentification rates of 12 and 0.9% for jets from charm quarks and light quarks or gluons, respectively.

Electron candidates are reconstructed by first matching reconstructed tracks to clusters of energy deposited in the ECAL. Selections based on the spatial distribution of the shower, track–cluster matching criteria, and consistency between the cluster energy and the track momentum are then used in the identification of electron candidates [46]. Muon candidates are reconstructed by requiring reconstructed tracks in the muon detector to be matched to the tracks found in the inner tracker [47]. We require the origin of electron and muon candidates to be consistent with the PV. Restrictions are imposed on the magnitude of the impact parameters of their tracks relative to the PV in the transverse plane ($d_{xy}$), and on the longitudinal displacement ($d_{z}$) of the point of closest approach. To ensure that electron or muon candidates are isolated from jet activity, we define a relative isolation quantity ($I_{\text {rel}}$) as the ratio of the scalar $p_{\mathrm {T}}$ sum of hadron and photon PF candidates, in an $\eta$-$\phi$ cone of radius 0.3 or 0.4 around the candidate electron or muon, to the candidate $p_{\mathrm {T}}$, requiring it to be below an upper bound appropriate for the selection. The quantity $I_{\text {rel}}$ is adjusted to account for the contributions of particles originating from pileup interactions. The electron and muon selection criteria applied in the analysis are the same as those described in Ref. [35].

The ${{\uptau }_{}^{}} _\mathrm {h}$ candidates are reconstructed using the CMS hadrons-plus-strips algorithm [48]. The constituents of the reconstructed jets are used to identify individual ${\uptau }_{}^{}$ lepton decay modes with one charged hadron and up to two neutral pions, or three charged hadrons. The ${{\uptau }_{}^{}} _\mathrm {h}$ candidate momentum is determined from the reconstructed visible ${\uptau }_{}^{}$ lepton decay products. The presence of extra particles within the jet that are incompatible with the reconstructed decay mode is used as a criterion to discriminate jets from ${{\uptau }_{}^{}} _\mathrm {h}$ decays. A multivariate-analysis (MVA) based discriminant [48], which contains isolation as well as lifetime information, is used to suppress the rate for quark and gluon jets to be misidentified as ${{\uptau }_{}^{}} _\mathrm {h}$ candidates. We employ a relaxed (“very loose”) working point of this discriminant as a preselection requirement for the ${{\uptau }_{}^{}} _\mathrm {h}$ candidates selected in the ${{\uptau }_{}^{}} _\mathrm {h} {{\uptau }_{}^{}} _\mathrm {h}$ analysis, as well as in the extrapolation used to estimate the contributions of events to the background in which quark or gluon jets are misidentified as ${{\uptau }_{}^{}} _\mathrm {h}$ candidates. This working point corresponds to an efficiency of $\approx$70% for a genuine ${{\uptau }_{}^{}} _\mathrm {h}$, and a misidentification rate of $\approx$1% for quark or gluon jets. A DNN is used to improve the discrimination of signal ${{\uptau }_{}^{}} _\mathrm {h}$ candidates from background, as discussed in more detail below. Two working points are used in the $\ell {{\uptau }_{}^{}} _\mathrm {h}$ analysis: a “very tight” working point for selecting signal ${{\uptau }_{}^{}} _\mathrm {h}$ candidates that provides stringent background rejection, and a “loose” working point for the extrapolation procedure to estimate the misidentified ${{\uptau }_{}^{}} _\mathrm {h}$ background that provides higher efficiency and less background rejection. These working points, respectively, typically have efficiencies close to 45 and 67% for a genuine ${{\uptau }_{}^{}} _\mathrm {h}$, with misidentification rates of $\approx$0.2 and 1% for quark or gluon jets. Electrons and muons misidentified as a ${{\uptau }_{}^{}} _\mathrm {h}$ are suppressed via criteria specifically developed for this purpose that are based on the consistency of information from the tracker, calorimeters, and muon detectors [48].

The dominant background in the ${{\uptau }_{}^{}} _\mathrm {h} {{\uptau }_{}^{}} _\mathrm {h}$ final state originates from misidentification of jets as ${{\uptau }_{}^{}} _\mathrm {h}$ candidates, mainly in SM events exclusively comprising jets produced through the strong interaction of quantum chromodynamics (QCD). These are referred to as QCD multijet events in what follows. To further improve the suppression of this background while retaining high signal efficiency, we have pursued a new approach for ${{\uptau }_{}^{}} _\mathrm {h}$ isolation in the ${{\uptau }_{}^{}} _\mathrm {h} {{\uptau }_{}^{}} _\mathrm {h}$ analysis that is based upon the application of a DNN that is fed information about the properties of PF candidates within an isolation cone with $\varDelta R < 0.5$ around the ${{\uptau }_{}^{}} _\mathrm {h}$ candidate. We refer to this as “Deep Particle Flow” (DeepPF) isolation. Charged PF candidates consistent with having originated from the PV, photon candidates, and neutral hadron candidates with $p_{\mathrm {T}} > 0.5$, 1, and 1.25$\,\text {GeV}$, respectively, provide the inputs to the DeepPF algorithm. The list of observables incorporated for each PF candidate includes its $p_{\mathrm {T}}$ relative to the ${{\uptau }_{}^{}} _\mathrm {h}$ jet, $\varDelta R$ between the candidate and ${{\uptau }_{}^{}} _\mathrm {h}$, particle type, track quality information, and $d_{xy}$, $d_{z}$ and their uncertainties, $\sigma (d_{xy})$ and $\sigma (d_{z})$. A convolutional DNN [49] is trained with simulated signal and background events. Signal ${{\uptau }_{}^{}} _\mathrm {h}$ candidates are those that are matched to generator-level ${\uptau }_{}^{}$ leptons from a mixture of processes that give rise to genuine ${\uptau }_{}^{}$ leptons. Background candidates that fail the matching are taken from simulated ${{\hbox {W}}_{}^{}}$+jets and QCD multijet events. The DeepPF discriminator value is obtained by averaging the DNN output with the nominal MVA-based discriminant described above. The working point for DeepPF isolation is chosen to maintain a constant efficiency of $\approx$50%, 56%, and 56% as a function of $p_{\mathrm {T}}$ for the three respective ${{\uptau }_{}^{}} _\mathrm {h}$ decay modes: one charged hadron, one charged hadron with neutral pions, and three charged hadrons. Since the ${{\uptau }_{}^{}} _\mathrm {h}$ candidate $p_{\mathrm {T}}$ distribution in signal events depends on the $\widetilde{\uptau }_{}^{}$ and ${\widetilde{\upchi }}_{1}^{0}$ masses, this choice of discriminator and working points allows us to maintain high efficiency for $\widetilde{\uptau }_{}^{}$ pair production signals under a large range of mass hypotheses. The overall misidentification rate for jets not originating from ${\uptau }_{}^{}$ leptons ranges from 0.15% to 0.4% depending on $p_{\mathrm {T}}$ and decay mode.

Significant contributions to the SM background originate from Drell–Yan+jets (DY+jets), ${{\hbox {W}}_{}^{}}$+jets, ${\hbox {t}}_{}^{}$ $\overline{{{\hbox {t}}_{}^{}}}_{}^{}$, and diboson processes, as well as from QCD multijet events, where DY corresponds to processes such as ${{\hbox {q}}_{}^{}} {\overline{\hbox {q}}_{}^{}} \rightarrow \ell ^{+}\ell ^{-}$. Smaller contributions arise from single top quark production and rare SM processes, such as triboson and Higgs boson production, and top quark pair production in association with vector bosons. We rely on a combination of measurements in data CRs and Monte Carlo (MC) simulation to estimate contributions of each source of background. The MC simulation is also used to model the signal.

The MadGraph 5_amc@nlo version 2.3.3 and 2.4.2 event generators [50] are used at leading order (LO) precision to generate simulated ${{\hbox {W}}_{}^{}}$+jets and DY+jets events with up to 4 additional partons for the analysis of 2016 and 2017 data, respectively. Exclusive event samples binned in jet multiplicity are used to enhance the statistical power of the simulation at higher values of jet multiplicity that are relevant to the phase space probed by this search. Production of top quark pairs, diboson and triboson events, and rare SM processes, such as single top quarks or top quark pairs associated with bosons, are generated at next-to-leading order (NLO) precision with MadGraph 5_amc@nlo and powhegv2 [51–54]. Showering and hadronization of partons are carried out using the pythia  8.205 and 8.230 packages [55] for the 2016 and 2017 analyses, respectively, while a detailed simulation of the CMS detector is based on the Geant4  [36] package. Finally, uncertainties in renormalization and factorization scale, and parton distribution functions (PDFs) have been obtained using the SysCalc package [56]. Models of direct $\widetilde{\uptau }_{}^{}$ pair production are generated with MadGraph 5_amc@nlo at LO precision up to the production of ${\uptau }_{}^{}$ leptons, with their decay modeled by pythia  8.212 and 8.230 for the analysis of 2016 and 2017 data, respectively. The CUETP8M1 [57] (CUETP8M2T4 [58] for ${\hbox {t}}_{}^{}$ $\overline{{{\hbox {t}}_{}^{}}}_{}^{}$) and CP5 [59] underlying-event tunes are used with pythia for the 2016 and 2017 analyses, respectively. The 2016 analysis uses the NNPDF3.0LO [60] set of PDFs in generating ${{\hbox {W}}_{}^{}}$+jets, DY+jets, and signal events, while the NNPDF3.0NLO PDFs are used for other processes. The NNPDF3.1NLO PDFs are used for all simulated events in the 2017 analysis.

Simulated events are reweighted to match the pileup profile observed in data. Differences between data and simulation in electron, muon, and ${{\uptau }_{}^{}} _\mathrm {h}$ identification and isolation efficiencies, jet, electron, muon, and ${{\uptau }_{}^{}} _\mathrm {h}$ energy scales, and ${\hbox {b}}_{}^{}$ tagging efficiency are taken into account by applying scale factors to the simulation. We improve the modeling of initial-state radiation (ISR) in simulated signal events by reweighting the $p_{\mathrm {T}} ^{\mathrm {ISR}}$ distribution, where $p_{\mathrm {T}} ^{\mathrm {ISR}}$ corresponds to the total transverse momentum of the system of SUSY particles. This reweighting procedure is based on studies of the $p_{\mathrm {T}}$ of ${\hbox {Z}}_{}^{}$ bosons [61]. The signal production cross sections are calculated at NLO using next-to-leading logarithmic (NLL) soft-gluon resummations [37]. The most precise calculated cross sections available are used to normalize the simulated SM background samples, often corresponding to next-to-next-to-leading order accuracy.

## Event selection

The search strategy in the ${{\uptau }_{}^{}} _\mathrm {h} {{\uptau }_{}^{}} _\mathrm {h}$ final state relies on a cut-and-count analysis based on the SRs described below in Sect. 4.1, while for the $\ell {{\uptau }_{}^{}} _\mathrm {h}$ final states we make use of BDTs to discriminate between signal and background as described in Sect. 4.2. The data used in this search are selected through triggers that require the presence of isolated electrons, muons, ${{\uptau }_{}^{}} _\mathrm {h}$ candidates, or $p_{\mathrm {T}} ^\text {miss}$. The data used for the ${{\uptau }_{}^{}} _\mathrm {h} {{\uptau }_{}^{}} _\mathrm {h}$ analysis are collected with two sets of triggers. Events with $p_{\mathrm {T}} ^\text {miss} < 200$ $\,\text {GeV}$ are selected using a trigger that requires the presence of two ${{\uptau }_{}^{}} _\mathrm {h}$ candidates, each with $p_{\mathrm {T}} >35$ and >40$\,\text {GeV}$ in 2016 and 2017 data, respectively. We gain up to 7% additional signal efficiency for events with $p_{\mathrm {T}} ^\text {miss} >200$ $\,\text {GeV}$ with the help of a trigger that requires the presence of substantial $p_{\mathrm {T}} ^\text {miss}$, with a threshold varying between 100 and 140$\,\text {GeV}$ during the 2016 and 2017 data-taking periods. For the ${{\hbox {e}}_{}^{}} {{\uptau }_{}^{}} _\mathrm {h}$ final state, the trigger relies on the presence of an isolated electron satisfying stringent identification criteria and passing $p_{\mathrm {T}} >25$ or >35$\,\text {GeV}$ in 2016 and 2017 data, respectively. For the ${{\upmu }_{}^{}} {{\uptau }_{}^{}} _\mathrm {h}$ final state, the trigger is based on the presence of an isolated muon with $p_{\mathrm {T}} >24$ and >27$\,\text {GeV}$ in 2016 and 2017 data, respectively. Trigger efficiencies are measured in data and simulation. In addition to corrections mentioned in Sect. 3, we apply scale factors to the simulation to account for any discrepancies in trigger efficiency with data. These scale factors are parameterized in the $p_{\mathrm {T}}$ and $\eta$ of the reconstructed electron, muon, or ${{\uptau }_{}^{}} _\mathrm {h}$ candidates, or the reconstructed $p_{\mathrm {T}} ^\text {miss}$ for events selected using $p_{\mathrm {T}} ^\text {miss}$ triggers.

### Event selection and search regions in the ${{\uptau }_{}^{}} _\mathrm {h} {{\uptau }_{}^{}} _\mathrm {h}$ final state

Beyond the trigger selection, the baseline event selection for the ${{\uptau }_{}^{}} _\mathrm {h} {{\uptau }_{}^{}} _\mathrm {h}$ analysis requires the presence of exactly two isolated ${{\uptau }_{}^{}} _\mathrm {h}$ candidates of opposite charge, satisfying the DeepPF selection described in Sect. 3, with $|\eta |<2.3$ and $p_{\mathrm {T}} > 40$ and >45$\,\text {GeV}$ in the 2016 and 2017 analysis, respectively, as well as no additional ${{\uptau }_{}^{}} _\mathrm {h}$ candidates with $p_{\mathrm {T}} > 30\,\text {GeV}$ satisfying the very loose working point of the MVA-based discriminant. We veto events with additional electrons or muons with $p_{\mathrm {T}} >20\,\text {GeV}$ and $|\eta | < 2.5$ or <2.4 for electrons and muons, respectively, and reject any events with a ${\hbox {b}}_{}^{}$-tagged jet to suppress top quark backgrounds. A requirement of $|\varDelta \phi ({{\uptau }_{}^{}} _\mathrm {h} ^{(1)},{{\uptau }_{}^{}} _\mathrm {h} ^{(2)}) | > 1.5$ helps to suppress the DY+jets background, while retaining high signal efficiency. Finally, we require $p_{\mathrm {T}} ^\text {miss} >50\,\text {GeV}$ to suppress the QCD multijet background.

The removal of low-$p_{\mathrm {T}}$ jets in the forward ECAL region from the ${\vec p}_{\mathrm {T}}^{\text {miss}}$ calculation in 2017 (see Sect. 3) causes the background originating from DY+jets and other sources to increase in the SRs, since events with low-$p_{\mathrm {T}}$ jet activity in that region are assigned larger values of reconstructed $p_{\mathrm {T}} ^\text {miss}$. We recover some of the corresponding loss in sensitivity in the 2017 analysis by placing an upper bound of 50$\,\text {GeV}$ on the scalar $p_{\mathrm {T}}$ sum of low-$p_{\mathrm {T}}$ jets excluded from the ${\vec p}_{\mathrm {T}}^{\text {miss}}$ calculation ($H_{\mathrm {T}} ^{\text {low}}$). This restriction reduces the impact of background events with significant low-$p_{\mathrm {T}}$ jet activity in the forward region, for which the $p_{\mathrm {T}} ^\text {miss}$ would be overestimated. To ensure that the efficiency of this requirement is correctly estimated in simulation, a ${{\hbox {Z}}_{}^{}} \rightarrow {{\upmu }_{}^{}} ^{+}{{\upmu }_{}^{}} ^{-}$ CR is used to extract correction factors for the $H_{\mathrm {T}} ^{\text {low}}$ distribution in simulation that account for discrepancies with the distribution observed in data. The correction factors range from 0.8 for $H_{\mathrm {T}} ^{\text {low}}<10\,\text {GeV}$ to 1.4 for $H_{\mathrm {T}} ^{\text {low}}>60\,\text {GeV}$. In addition, to avoid effects related to jet mismeasurement that can contribute to spurious $p_{\mathrm {T}} ^\text {miss}$, we require the ${\vec p}_{\mathrm {T}}^{\text {miss}}$ to have a minimum separation of 0.25 in $|\varDelta \phi |$ from jets with $p_{\mathrm {T}} > 30\,\text {GeV}$ and $|\eta | < 2.4$, as well as from those with uncorrected $p_{\mathrm {T}} > 50\,\text {GeV}$ in the region $2.4< |\eta | < 3.14$.

Events satisfying the baseline selection criteria are subdivided into exclusive SRs using several discriminants. To improve the discrimination of signal from SM background, we take advantage of the expected presence of two ${\widetilde{\upchi }}_{1}^{0}$ in the final state of signal events and their contribution to $p_{\mathrm {T}} ^\text {miss}$. Their presence skews the correlations between ${\vec p}_{\mathrm {T}}^{\text {miss}}$ and the reconstructed leptons to be different from background processes, even for those backgrounds with genuine $p_{\mathrm {T}} ^\text {miss}$. These differences can be exploited by mass observables calculated from the reconstructed lepton transverse momenta and ${\vec p}_{\mathrm {T}}^{\text {miss}}$ to provide discrimination of signal from background. For a particle decaying to a visible and an invisible particle, the transverse mass ($m_{\mathrm {T}}$) calculated from the ${\vec p}_{\mathrm {T}}$ of the visible decay products should have a kinematic endpoint at the mass of the parent particle. Assuming that the $p_{\mathrm {T}} ^\text {miss}$ corresponds to the $p_{\mathrm {T}}$ of the invisible particle, we calculate the $m_{\mathrm {T}}$ observable for the visible particle q and the invisible particle as follows:

1 $$\begin{aligned} m_{\mathrm {T}} (\mathrm {q}, {\vec p}_{\mathrm {T}}^{\text {miss}}) \equiv \sqrt{2 p_{\mathrm {T}} ^{\mathrm {q}} p_{\mathrm {T}} ^\text {miss} [1 - \cos \varDelta \phi ({\vec p}_{\mathrm {T}} ^{\mathrm {q}}, {\vec p}_{\mathrm {T}}^{\text {miss}})]}. \end{aligned}$$

We use as a discriminant the sum of the transverse masses calculated for each ${{\uptau }_{}^{}} _\mathrm {h}$ with $p_{\mathrm {T}} ^\text {miss}$, $\varSigma m_{\mathrm {T}}$, given by

2 $$\begin{aligned} \varSigma m_{\mathrm {T}} = m_{\mathrm {T}} ({{{\uptau }_{}^{}} _\mathrm {h} ^{(1)},{\vec p}_{\mathrm {T}}^{\text {miss}}}) + m_{\mathrm {T}} ({{{\uptau }_{}^{}} _\mathrm {h} ^{(2)},{\vec p}_{\mathrm {T}}^{\text {miss}}}). \end{aligned}$$

Another variable found to be useful in the discrimination of signal from background is the “stransverse mass” $m_{\mathrm {T2}}$  [62–64]. This mass variable is a generalization of $m_{\mathrm {T}}$ in the case of multiple invisible particles. It serves as an estimator of the mass of pair-produced particles when both particles decay to a final state containing the same invisible particle. It is given by:

3 $$\begin{aligned} m_{\mathrm {T2}} = \min _{{\vec p}_{\mathrm {T}} ^{\mathrm {X}(1)} + {\vec p}_{\mathrm {T}} ^{\mathrm {X}(2)} = {\vec p}_{\mathrm {T}}^{\text {miss}}} \left[ \max \left( m_{\mathrm {T}} ^{(1)} , m_{\mathrm {T}} ^{(2)} \right) \right] , \end{aligned}$$

where ${\vec p}_{\mathrm {T}} ^{\mathrm {X}(i)}$ (with *i*=1, 2) are the unknown transverse momenta of the two undetected particles, X(1) and X(2), corresponding to the neutralinos in our signal models, and $m_{\mathrm {T}} ^{(i)}$ are the transverse masses obtained by pairing either of the two invisible particles with one of the two leptons. The minimization ($\min$) is over the possible momenta of the invisible particles, taken to be massless, which are constrained to add up to the ${\vec p}_{\mathrm {T}}^{\text {miss}}$ in the event. For direct $\widetilde{\uptau }_{}^{}$ pair production, with each $\widetilde{\uptau }_{}^{}$ decaying to a ${\uptau }_{}^{}$ lepton and a ${\widetilde{\upchi }}_{1}^{0}$, $m_{\mathrm {T2}}$ should be correlated with the mass difference between the $\widetilde{\uptau }_{}^{}$ and ${\widetilde{\upchi }}_{1}^{0}$. A large value of $m_{\mathrm {T2}}$ is thus common in signal events for models with larger $\widetilde{\uptau }_{}^{}$ masses and relatively rare in SM background events.

**Fig. 2:**
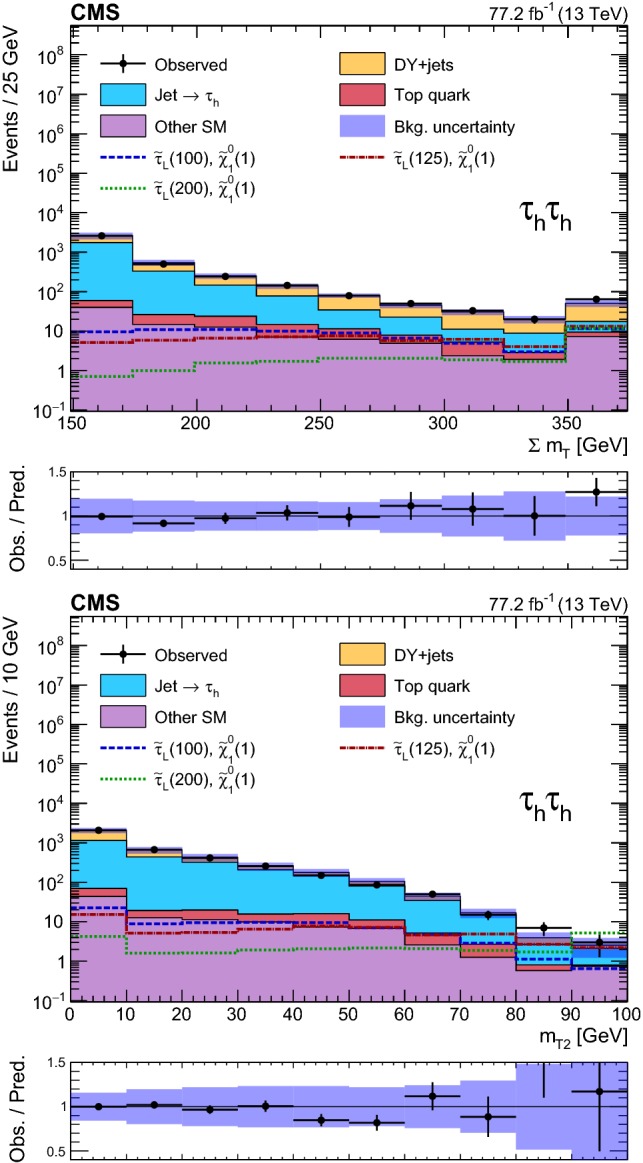
Distributions in $\varSigma m_{\mathrm {T}}$ (upper) and $m_{\mathrm {T2}}$ (lower) for events in the combined 2016 and 2017 data sets passing the baseline selection in the ${{\uptau }_{}^{}} _\mathrm {h} {{\uptau }_{}^{}} _\mathrm {h}$ final state, along with the corresponding prediction for the SM background and three benchmark models for ${\widetilde{\uptau }}_{\mathrm {L}}$ pair production with $m({\widetilde{\uptau }}_{\mathrm {L}})=100$, 125, and 200$\,\text {GeV}$, $m({{\widetilde{\upchi }}_{1}^{0}})=1\,\text {GeV}$. The numbers within parentheses in the legend correspond to the masses of the ${\widetilde{\uptau }}_{\mathrm {L}}$ and ${\widetilde{\upchi }}_{1}^{0}$ in $\,\text {GeV}$. The last bin includes overflow events in each case. The shaded uncertainty bands represent the combined statistical and systematic uncertainties in the background

The SR definitions for the ${{\uptau }_{}^{}} _\mathrm {h} {{\uptau }_{}^{}} _\mathrm {h}$ analysis, shown in Table 1, are based on a cut-and-count analysis of the sample satisfying the baseline selections. The regions are defined through criteria imposed on $m_{\mathrm {T2}}$, $\varSigma m_{\mathrm {T}}$, and the number of reconstructed jets in an event, $N_{\text {j}}$. The $\varSigma m_{\mathrm {T}}$ and $m_{\mathrm {T2}}$ distributions of events in the ${{\uptau }_{}^{}} _\mathrm {h} {{\uptau }_{}^{}} _\mathrm {h}$ final state surviving the baseline selections are shown in Fig. 2. The distributions obtained for 2016 and 2017 data are combined. Separate sets of simulated events are used to model signal and background events in 2016 and 2017 data using the methods described in Sect. 3. In all distributions, the last bin includes overflow events. After applying a minimum requirement of $m_{\mathrm {T2}} >25\,\text {GeV}$ in all SRs, we subdivide events into low (25–50$\,\text {GeV}$) and high (${>}50\,\text {GeV}$) $m_{\mathrm {T2}}$ regions, to improve the sensitivity to lower and higher $\widetilde{\uptau }_{}^{}$ mass signals, respectively. For each $m_{\mathrm {T2}}$ region, the $\varSigma m_{\mathrm {T}}$ distribution is exploited to provide sensitivity for a large range of $\widetilde{\uptau }_{}^{}$ mass signals. We define three bins in $\varSigma m_{\mathrm {T}}$: 200–250, 250–300, and >300$\,\text {GeV}$. Finally, we subdivide events in each $m_{\mathrm {T2}}$ and $\varSigma m_{\mathrm {T}}$ region into the categories $N_{\text {j}} = 0$ and $N_{\text {j}} \ge 1$. This binning is beneficial as background events passing the SR kinematic selections are largely characterized by additional jet activity, while signal contains very few additional jets. The 0-jet category therefore provides nearly background-free SRs. However, we retain the SRs with $N_{\text {j}} \ge 1$ that are also expected to contain signal events with ISR or pileup jets.

**Table 1:** Ranges in $m_{\mathrm {T2}}$, $\varSigma m_{\mathrm {T}}$, and $N_{\text {j}}$ used to define the SRs used in the ${{\uptau }_{}^{}} _\mathrm {h} {{\uptau }_{}^{}} _\mathrm {h}$ analysis

$m_{\mathrm {T2}}$ [$\text {GeV}$ ]	25–50						>50					
$\varSigma m_{\mathrm {T}}$ [$\text {GeV}$ ]	200–250		250–300		>300		200–250		250–300		>300	
$N_{\text {j}}$	0	$\ge$1	0	$\ge$1	0	$\ge$1	0	$\ge$1	0	$\ge$1	0	$\ge$1

### Event selection in the $\ell {{\uptau }_{}^{}} _\mathrm {h}$ final states

The baseline event selections for the $\ell {{\uptau }_{}^{}} _\mathrm {h}$ analyses require either an electron with $p_{\mathrm {T}} > 26\,(35)$ $\,\text {GeV}$ and $|\eta | < 2.1$ or a muon with $p_{\mathrm {T}} > 25\,(28)$ $\,\text {GeV}$ and $|\eta |<2.4$ for the 2016 (2017) data, and a ${{\uptau }_{}^{}} _\mathrm {h}$ candidate with $p_{\mathrm {T}} > 30$ $\,\text {GeV}$ and $|\eta | < 2.3$. Electrons, muons, and ${{\uptau }_{}^{}} _\mathrm {h}$ candidates are required to have $|d_{z} | < 0.2\,\text {cm}$, and electrons and muons are also required to have $|d_{xy} | < 0.045\,\text {cm}$. Electrons and muons have to satisfy $I_{\text {rel}} <0.15$ and <0.1, respectively. Backgrounds from ${\hbox {t}}_{}^{}$ $\overline{{{\hbox {t}}_{}^{}}}_{}^{}$ and ${{\hbox {W}}_{}^{}}$+jets are greatly reduced by vetoing events that contain jets with $p_{\mathrm {T}} > 20\,\text {GeV}$. Events from the ${{\hbox {W}}_{}^{}}$+jets background are further reduced by requiring the transverse mass $m_{\mathrm {T}} (\ell , {\vec p}_{\mathrm {T}}^{\text {miss}})$, calculated using the electron or muon momentum vector and ${\vec p}_{\mathrm {T}}^{\text {miss}}$, to be between 20 and 60$\,\text {GeV}$ or above 120$\,\text {GeV}$. A significant background from DY+jets events is reduced by requiring the invariant mass of the electron or muon and the ${{\uptau }_{}^{}} _\mathrm {h}$, $m_{\ell {{\uptau }_{}^{}} _\mathrm {h}}$ to be above 50$\,\text {GeV}$. To reduce background from QCD multijet events, we require $2.0< \varDelta R(\ell , {{\uptau }_{}^{}} _\mathrm {h}) <3.5$.

With these preselection criteria in place, we train several BDTs corresponding to different signal hypotheses to classify signal and background events. The input variables are the $p_{\mathrm {T}}$ of the electron or muon, the $p_{\mathrm {T}}$ of the ${{\uptau }_{}^{}} _\mathrm {h}$ candidate, $p_{\mathrm {T}} ^\text {miss}$, $m_{\mathrm {T}} (\ell , {\vec p}_{\mathrm {T}}^{\text {miss}})$, $\varDelta \eta (\ell ,{{\uptau }_{}^{}} _\mathrm {h})$, $\varDelta \phi (\ell , {\vec p}_{\mathrm {T}}^{\text {miss}})$, $\varDelta \phi ({{\uptau }_{}^{}} _\mathrm {h}, {\vec p}_{\mathrm {T}}^{\text {miss}})$, $\varDelta R(\ell , {{\uptau }_{}^{}} _\mathrm {h})$, $m(\ell {{\uptau }_{}^{}} _\mathrm {h})$, and $m_{\mathrm {T}} ^{\text {tot}}\equiv \sqrt{\smash [b]{m_{\mathrm {T}} ^2(\ell , {\vec p}_{\mathrm {T}}^{\text {miss}})+m_{\mathrm {T}} ^2({{\uptau }_{}^{}} _\mathrm {h}, {\vec p}_{\mathrm {T}}^{\text {miss}})}}$. We also include $m_{\mathrm {T2}}$ and the contransverse mass ($m_{\mathrm {CT}}$) [65, 66], computed from the visible decay products and defined as

4 $$\begin{aligned} m_{\mathrm {CT}} \equiv \sqrt{2 p_{\mathrm {T}} ^{\ell } p_{\mathrm {T}} ^{{{\uptau }_{}^{}} _\mathrm {h}} [1 + \cos \varDelta \phi (\ell ,{{\uptau }_{}^{}} _\mathrm {h})]}. \end{aligned}$$

**Fig. 3:**
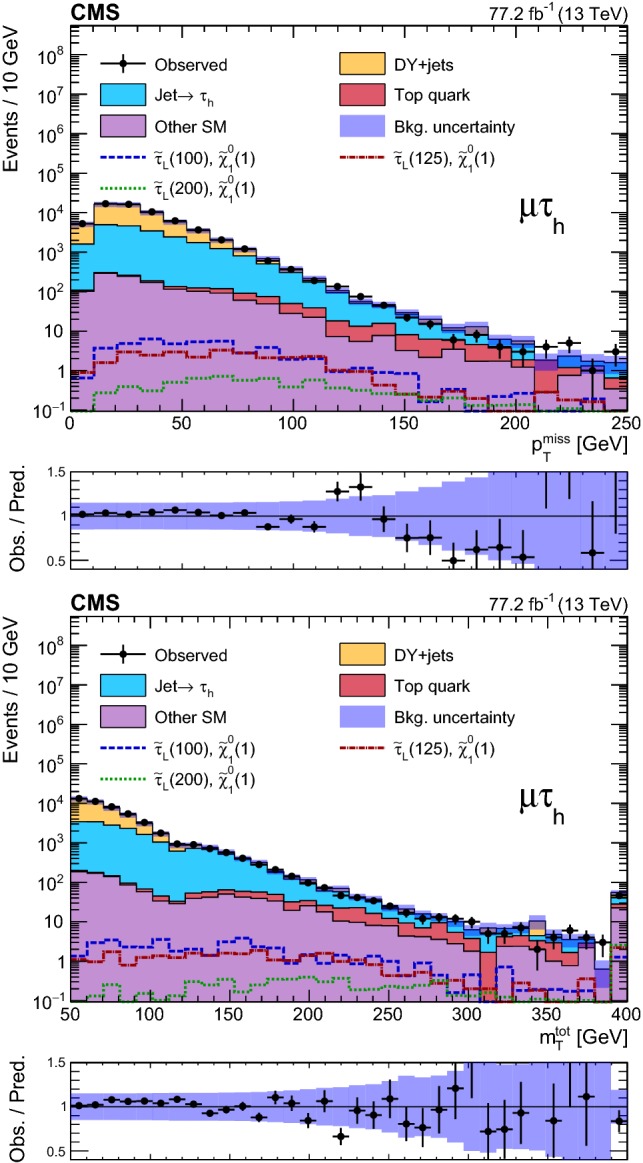
Distributions in $p_{\mathrm {T}} ^\text {miss}$ (upper) and $m_{\mathrm {T}} ^{\text {tot}}$ (lower) for events in the combined 2016 and 2017 data passing the baseline selections in the ${{\upmu }_{}^{}} {{\uptau }_{}^{}} _\mathrm {h}$ final state, along with the corresponding prediction for SM background and three benchmark models of ${\widetilde{\uptau }}_{\mathrm {L}}$ pair production with $m({\widetilde{\uptau }}_{\mathrm {L}})=100$, 125, and 200$\,\text {GeV}$ and $m({{\widetilde{\upchi }}_{1}^{0}})=1\,\text {GeV}$. The numbers within parentheses in the legend correspond to the masses of the ${\widetilde{\uptau }}_{\mathrm {L}}$ and ${\widetilde{\upchi }}_{1}^{0}$ in $\,\text {GeV}$. The last bin includes overflow events in each case. The shaded uncertainty bands represent the combined statistical and average systematic uncertainties in the background

For signal events, $m_{\mathrm {CT}}$ is expected to have an endpoint near ${(m({\widetilde{\uptau }_{}^{}})^2-m({{\widetilde{\upchi }}_{1}^{0}})^2)}/m({\widetilde{\uptau }_{}^{}})$. Finally, we include the variable $D_{\zeta } ={\vec p}_{\mathrm {T}}^{\text {miss}} \cdot \vec \zeta - 0.85 ({\vec p}_{\mathrm {T}} ^{\ell }+{\vec p}_{\mathrm {T}} ^{{{\uptau }_{}^{}} _\mathrm {h}}) \cdot \vec \zeta$, with $\vec {\zeta }$ being the bisector of the directions of the transverse momenta of the electron or muon and the ${{\uptau }_{}^{}} _\mathrm {h}$ candidate [67, 68]. The value of 0.85 reflects an optimization to efficiently distinguish DY+jets events from other backgrounds and the signal. Figure 3 shows the distributions of events passing the baseline selections in the ${{\upmu }_{}^{}} {{\uptau }_{}^{}} _\mathrm {h}$ final state in two of the BDT input variables that provide the highest discriminating power, $p_{\mathrm {T}} ^\text {miss}$ and $m_{\mathrm {T}} ^{\text {tot}}$. The distributions observed in the ${{\hbox {e}}_{}^{}} {{\uptau }_{}^{}} _\mathrm {h}$ final state are similar.

Since the signal kinematics depend on mass, we train BDTs for signals with $\widetilde{\uptau }_{}^{}$ masses of 100, 150, and 200$\,\text {GeV}$. In all cases we use a ${\widetilde{\upchi }}_{1}^{0}$ mass of 1$\,\text {GeV}$. As the results of the training depend critically on the number of input events, we relax the ${{\uptau }_{}^{}} _\mathrm {h}$ MVA-based isolation criteria and reduce the $p_{\mathrm {T}}$ threshold for the ${{\uptau }_{}^{}} _\mathrm {h}$ to 20$\,\text {GeV}$ for the training sample in order to increase the number of training and test events. The “very tight” isolation and a $p_{\mathrm {T}}$ threshold of 30$\,\text {GeV}$ for the ${{\uptau }_{}^{}} _\mathrm {h}$ are applied in the final analysis. For a given signal hypothesis, we choose the BDT trained with the same $\widetilde{\uptau }_{}^{}$ mass for models with $\widetilde{\uptau }_{}^{}$ masses of 100, 150, and 200$\,\text {GeV}$, or the one that provides optimal sensitivity for models with other $\widetilde{\uptau }_{}^{}$ mass values. For signal models with $\widetilde{\uptau }_{}^{}$ masses of 90 and 125$\,\text {GeV}$, we use the BDT trained for $m({\widetilde{\uptau }_{}^{}})=100\,\text {GeV}$, while for those with a $\widetilde{\uptau }_{}^{}$ mass of 175$\,\text {GeV}$, we use the BDT trained for $m({\widetilde{\uptau }_{}^{}})=200\,\text {GeV}$. While signal events are largely expected to have high BDT output values, we include the full BDT distribution in a binned fit for the statistical interpretation of the analysis as described in Sect. 7. The binning is chosen to optimize signal significance.

## Background estimation

Our most significant backgrounds are from DY+jets, ${{\hbox {W}}_{}^{}}$+jets, QCD multijet, ${\hbox {t}}_{}^{}$ $\overline{{{\hbox {t}}_{}^{}}}_{}^{}$, and diboson processes. They have relative contributions that vary with final state. For the ${{\uptau }_{}^{}} _\mathrm {h} {{\uptau }_{}^{}} _\mathrm {h}$ final state, the dominant background arises from the misidentification of jets as ${{\uptau }_{}^{}} _\mathrm {h}$ candidates in QCD multijet and ${{\hbox {W}}_{}^{}}$+jets events, constituting $\approx$65% of background after the baseline selection. For the $\ell {{\uptau }_{}^{}} _\mathrm {h}$ final states after the baseline selection, the main backgrounds are from DY+jets ($\approx$50%), ${{\hbox {W}}_{}^{}}$+jets ($\approx$30%), and QCD multijet ($\approx$10%) events. The DY+jets contribution, which is also a major background in the ${{\uptau }_{}^{}} _\mathrm {h} {{\uptau }_{}^{}} _\mathrm {h}$ final state ($\approx$20%), usually consists of events with two prompt ${\uptau }_{}^{}$ leptons. This background is determined with simulation samples after applying corrections to match the normalization and to be consistent with variable distributions in collider data. The ${{\hbox {W}}_{}^{}}$+jets and QCD multijet backgrounds usually contain one or more jets misidentified as ${{\uptau }_{}^{}} _\mathrm {h}$ and their contributions are determined via methods that rely on data. Finally, we have smaller contributions from other SM processes such as the production of Higgs bosons, dibosons, and top quark pairs with or without vector bosons. These are estimated via MC simulation with appropriate correction factors applied as described in Sect. 3. For the $\ell {{\uptau }_{}^{}} _\mathrm {h}$ analyses, dedicated CRs that are each enriched in one of the major background processes are used to validate the modeling of the BDT distribution and to extract uncertainties that are used to account for any potential mismodeling of the distributions in simulation. These CRs are described in the following subsections below.

### Estimation of background from misidentified jets

#### Misidentified jets in the ${{\uptau }_{}^{}} _\mathrm {h} {{\uptau }_{}^{}} _\mathrm {h}$ final state

After requiring two ${{\uptau }_{}^{}} _\mathrm {h}$ candidates with high $p_{\mathrm {T}}$, events with misidentified ${{\uptau }_{}^{}} _\mathrm {h}$ candidates are the dominant background in the ${{\uptau }_{}^{}} _\mathrm {h} {{\uptau }_{}^{}} _\mathrm {h}$ final state. This background, which originates predominantly from QCD multijet and ${{\hbox {W}}_{}^{}}$+jets production, is predicted by extrapolating the event count in a data sample selected with a relaxed isolation requirement into the SR. The fraction of non-prompt or misidentified ${{\uptau }_{}^{}} _\mathrm {h}$ candidates selected with the very loose MVA-based isolation working point that also pass the tight DeepPF isolation requirement is measured in a QCD multijet-enriched sample of same-charge ${{\uptau }_{}^{}} _\mathrm {h} {{\uptau }_{}^{}} _\mathrm {h}$ events. The same-charge ${{\uptau }_{}^{}} _\mathrm {h} {{\uptau }_{}^{}} _\mathrm {h}$ events are collected with the same ${{\uptau }_{}^{}} _\mathrm {h} {{\uptau }_{}^{}} _\mathrm {h}$ trigger as opposite-charge ${{\uptau }_{}^{}} _\mathrm {h} {{\uptau }_{}^{}} _\mathrm {h}$ events to avoid additional trigger-related biases. We also require $m_{\mathrm {T2}}$ to be low (<40$\,\text {GeV}$) to reduce potential contributions from signal events. We find that roughly 20% of the same-charge events with misidentified ${{\uptau }_{}^{}} _\mathrm {h}$ candidates selected with very loose isolation also pass the tight isolation requirement. However, the rate depends on the $p_{\mathrm {T}}$ and decay mode (one- or three-prongs) of the ${{\uptau }_{}^{}} _\mathrm {h}$ candidate, as well as the jet flavor, i.e., whether the misidentified jet originates from the hadronization of light-flavor quarks, heavy-flavor quarks, or gluons. The ${{\uptau }_{}^{}} _\mathrm {h}$ misidentification rate is therefore measured in bins of $p_{\mathrm {T}}$ and decay mode to mitigate the dependence on these factors. The measurement is also binned in the number of primary vertices ($N_{\mathrm {PV}}$) to capture the effects of pileup. From studies performed with MC simulation samples, a systematic uncertainty of $\approx$30% is assigned to account for the dependence of the misidentification rate on jet flavor.

Since the isolation efficiency for prompt ${{\uptau }_{}^{}} _\mathrm {h}$ candidates is only around 70–80%, processes containing genuine ${{\uptau }_{}^{}} _\mathrm {h}$ candidates can enter the sideband regions in events that are selected with the relaxed isolation requirement. To take this into account when calculating the final background estimate, we define three categories of events with at least two loosely isolated ${{\uptau }_{}^{}} _\mathrm {h}$ candidates: (1) events in which both ${{\uptau }_{}^{}} _\mathrm {h}$ candidates pass the tight DeepPF isolation requirement, (2) events in which one passes and one fails the tight isolation requirement, and (3) events in which both ${{\uptau }_{}^{}} _\mathrm {h}$ candidates fail the tight isolation requirement. We then equate the count of events in each of these three event categories to the sum of expected counts for the events with two prompt ${{\uptau }_{}^{}} _\mathrm {h}$ candidates, two jets misidentified as ${{\uptau }_{}^{}} _\mathrm {h}$ candidates, or one prompt ${{\uptau }_{}^{}} _\mathrm {h}$ candidate and one jet misidentified as a ${{\uptau }_{}^{}} _\mathrm {h}$ candidate, that contribute to each category. The contributions from backgrounds with one or two jets misidentified as ${{\uptau }_{}^{}} _\mathrm {h}$ candidates in the SRs are then determined analytically by solving a set of linear equations.

#### Misidentified jets in the ${{\hbox {e}}_{}^{}} {{\uptau }_{}^{}} _\mathrm {h}$ and ${{\upmu }_{}^{}} {{\uptau }_{}^{}} _\mathrm {h}$ final states

The misidentification of jets as ${{\uptau }_{}^{}} _\mathrm {h}$ candidates also gives rise to a major source of background in the ${{\hbox {e}}_{}^{}} {{\uptau }_{}^{}} _\mathrm {h}$ and ${{\upmu }_{}^{}} {{\uptau }_{}^{}} _\mathrm {h}$ final states that arises mainly from ${{\hbox {W}}_{}^{}}$+jets events with leptonic ${\hbox {W}}_{}^{}$ boson decays. We estimate this background from a sideband region in data selected using the SR selection criteria, with the exception that the ${{\uptau }_{}^{}} _\mathrm {h}$ candidates are required to satisfy the loose isolation working point and not the very tight working point. A transfer factor for the extrapolation of event counts from this ${{\uptau }_{}^{}} _\mathrm {h}$-isolation range into the tight isolation range of the SR is determined with a ${{\hbox {W}}_{}^{}}$+jets CR selected from events with one muon and at least one ${{\uptau }_{}^{}} _\mathrm {h}$ candidate that passes the loose isolation requirement. In events with more than one ${{\uptau }_{}^{}} _\mathrm {h}$ candidate, the candidate with the highest value of the MVA-based isolation discriminant is used. To increase the purity of ${{\hbox {W}}_{}^{}}$+jets events in this region, we reduce the contribution from ${\hbox {t}}_{}^{}$ $\overline{{{\hbox {t}}_{}^{}}}_{}^{}$ and QCD multijet events by requiring $60< m_{\mathrm {T}} (\ell ,{\vec p}_{\mathrm {T}}^{\text {miss}}) < 120\,\text {GeV}$, $p_{\mathrm {T}} ^\text {miss} > 40\,\text {GeV}$, no more than two jets, and an azimuthal separation of at least 2.5 radians between any jet and the ${\hbox {W}}_{}^{}$ boson reconstructed from the muon and ${\vec p}_{\mathrm {T}}^{\text {miss}}$ ($\varDelta \phi ({{\hbox {W}}_{}^{}},\text {jet})>2.5$). We also reject events with additional electrons or muons satisfying looser identification criteria. The remaining sample has an expected purity of $\approx$85% for ${{\hbox {W}}_{}^{}}$+jets events. The transfer factor, *R*, is then determined from this control sample after subtracting the remaining non-${{\hbox {W}}_{}^{}}$+jets background contributions estimated from simulation, as follows:

5 $$\begin{aligned} R = \frac{N^{\mathrm {CR}}_{\text {data}}({\mathrm {VT}})-N^{\mathrm {CR}}_{\text {MC no }{{\hbox {W}}_{}^{}}}({\mathrm {VT}})}{N^{\mathrm {CR}}_{\text {data}} (\mathrm {L}\overline{\mathrm {VT}})-N^{\mathrm {CR}}_{\text {MC no }{{\hbox {W}}_{}^{}}} (\mathrm {L}\overline{\mathrm {VT}})}, \end{aligned}$$

where $N^{\mathrm {CR}}_{\text {data}}$ corresponds to the number of events in the CR in data. The parenthetical argument $\mathrm {VT}$ denotes events in which the ${{\uptau }_{}^{}} _\mathrm {h}$ candidate satisfies the very tight isolation working point, while $\mathrm {L}\overline{\mathrm {VT}}$ denotes those that satisfy the loose, but not the very tight requirement. Transfer factors are determined separately in bins of $p_{\mathrm {T}}$ and $\eta$ of ${{\uptau }_{}^{}} _\mathrm {h}$ candidates in order to achieve an accurate description of the background.

The contribution of the background originating from a jet misidentified as a ${{\uptau }_{}^{}} _\mathrm {h}$ candidate in the SR is then determined from the corresponding sideband in data:

6 $$\begin{aligned} N(\text {jet} \rightarrow {{\uptau }_{}^{}} _\mathrm {h}) = R \, (N^{\text {sideband}}_{\text {data}} - N^{\text {sideband}}_{\mathrm {MC}, {{\uptau }_{}^{}}}), \end{aligned}$$

where $N^{\text {sideband}}_{\text {data}}$ is the number of events in the sideband in data, from which $N^{\text {sideband}}_{\mathrm {MC}, {{\uptau }_{}^{}}}$, the number with genuine ${\uptau }_{}^{}$ leptons as estimated with MC simulation by generator-level matching, is subtracted. We validate the estimation of jets misidentified as ${{\uptau }_{}^{}} _\mathrm {h}$ in a CR requiring $60< m_{\mathrm {T}} (\ell ,{\vec p}_{\mathrm {T}}^{\text {miss}}) < 120\,\text {GeV}$ and $\varDelta \phi ({{\hbox {W}}_{}^{}},\text {jet})<2.5$ to ensure that the region is independent of the region described above that is used to estimate the background.

### Estimation of background from Drell–Yan+jets

**Fig. 4:**
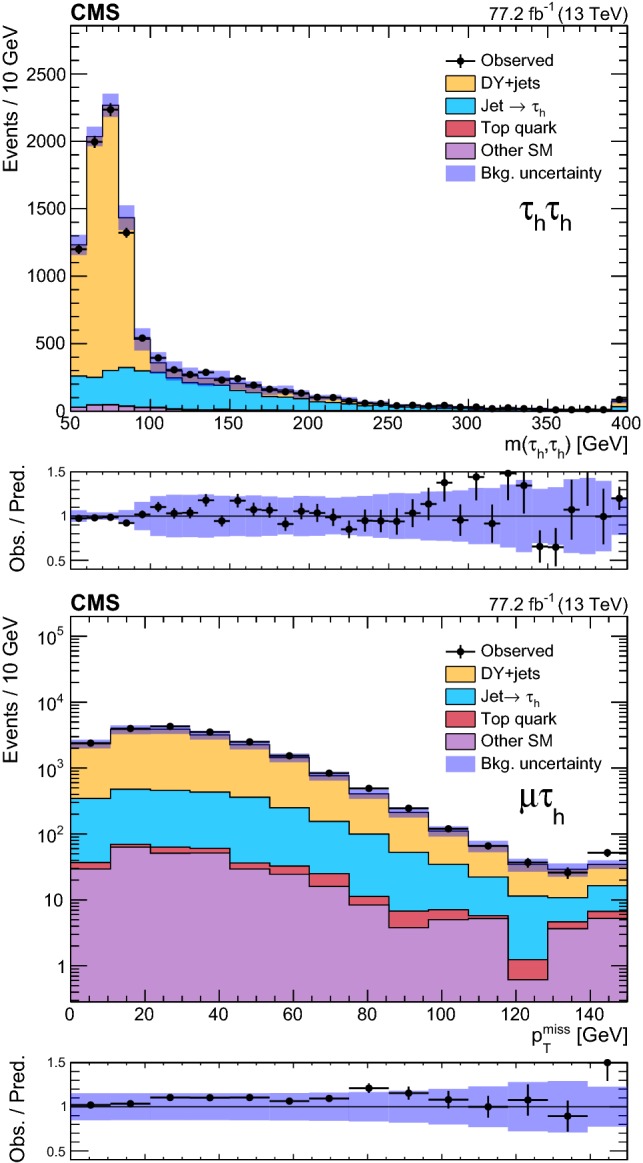
Visible-mass spectra of ${\uptau }_{}^{}$ lepton pairs in ${{\uptau }_{}^{}} _\mathrm {h} {{\uptau }_{}^{}} _\mathrm {h}$ events (upper) and $p_{\mathrm {T}} ^\text {miss}$ distribution in ${{\upmu }_{}^{}} {{\uptau }_{}^{}} _\mathrm {h}$ events (lower) in data and the corresponding prediction for SM background in the combined 2016 and 2017 DY+jets validation regions. The last bin includes overflow events in each case. The shaded uncertainty band represents the statistical and systematic uncertainties in the background prediction. For the ${{\upmu }_{}^{}} {{\uptau }_{}^{}} _\mathrm {h}$ distribution, the systematic uncertainty included in each bin corresponds to a single common average value

The DY+jets background comes primarily from ${{\hbox {Z}}_{}^{}} \rightarrow {{\uptau }_{}^{}} ^{+}{{\uptau }_{}^{}} ^{-}$ decays. We estimate this contribution via simulation, after applying corrections based on CRs in data. Mismodeling of the ${\hbox {Z}}_{}^{}$ boson mass or $p_{\mathrm {T}}$ distribution in simulation can lead to significant differences between data and simulation in kinematic discriminant distributions, especially when considering the large values of these variables that are relevant for the ${{\uptau }_{}^{}} _\mathrm {h} {{\uptau }_{}^{}} _\mathrm {h}$ SRs. We therefore use a high-purity ${{\hbox {Z}}_{}^{}} \rightarrow {{\upmu }_{}^{}} ^{+}{{\upmu }_{}^{}} ^{-}$ CR to compare the dimuon mass and $p_{\mathrm {T}}$ spectra between data and simulation and use the observed differences to correct the simulation in the SRs with weights parameterized by generator-level ${\hbox {Z}}_{}^{}$ boson mass and $p_{\mathrm {T}}$. The correction factors range up to 30% for high-mass and high-$p_{\mathrm {T}}$ values. Because these factors are intended to compensate for missing higher-order effects in the simulation, we assign the differences between the generator-level ${\hbox {Z}}_{}^{}$ boson mass and $p_{\mathrm {T}}$ distributions in LO and NLO simulated events as systematic uncertainties. The differences between data and simulation are taken into account through the use of scale factors, as described in Sect. 3. The uncertainties in these corrections are propagated to the final background estimate. The corrected simulation is validated in the ${{\uptau }_{}^{}} _\mathrm {h} {{\uptau }_{}^{}} _\mathrm {h}$ final state using a ${{\hbox {Z}}_{}^{}} \rightarrow {{\uptau }_{}^{}} ^{+}{{\uptau }_{}^{}} ^{-}$ CR selected by inverting the $m_{\mathrm {T2}}$ and $\varSigma m_{\mathrm {T}}$ requirements used to define the SRs. In addition, requiring a $p_{\mathrm {T}}$ of at least 50$\,\text {GeV}$ for the ${{\uptau }_{}^{}} _\mathrm {h} {{\uptau }_{}^{}} _\mathrm {h}$ system reduces the QCD multijet background and improves the purity of this CR. This choice makes it possible to increase the statistical power of this region by removing the $p_{\mathrm {T}} ^\text {miss} >50\,\text {GeV}$ requirement. The visible mass distribution of the ${{\uptau }_{}^{}} _\mathrm {h} {{\uptau }_{}^{}} _\mathrm {h}$ system shown in Fig. 4 (upper) demonstrates that the corrected simulation agrees with the data within experimental uncertainties.

For the analysis in the $\ell {{\uptau }_{}^{}} _\mathrm {h}$ final states, a normalization scale factor, as well as corrections to the $p_{\mathrm {T}}$ distribution of the ${\hbox {Z}}_{}^{}$ boson in simulation are obtained from a very pure ${{\hbox {Z}}_{}^{}} \rightarrow {{\upmu }_{}^{}} ^{+}{{\upmu }_{}^{}} ^{-}$ CR in data. These events are selected by requiring two isolated muons and no additional leptons, at most one jet, no ${\hbox {b}}_{}^{}$-tagged jets, and a dimuon mass in a window of 75–105$\,\text {GeV}$, to increase the probability to >99% that they originate from ${{\hbox {Z}}_{}^{}} \rightarrow {{\upmu }_{}^{}} ^{+}{{\upmu }_{}^{}} ^{-}$ decays. After subtracting all other contributions estimated from simulation, a normalization scale factor of $0.96\pm 0.05$, which is compatible with unity, is extracted from the ratio of data to simulated events. The uncertainty in the scale factor is determined by varying systematic uncertainties associated with objects such as the muon efficiency and jet energy uncertainties.

To validate the DY+jets background prediction in the $\ell {{\uptau }_{}^{}} _\mathrm {h}$ analyses, we construct a CR in ${{\upmu }_{}^{}} {{\uptau }_{}^{}} _\mathrm {h}$ events with $m_{\mathrm {T}} ({{\upmu }_{}^{}},{\vec p}_{\mathrm {T}}^{\text {miss}}) < 20\,\text {GeV}$, $50< m({{\upmu }_{}^{}} {{\uptau }_{}^{}} _\mathrm {h} ) < 80\,\text {GeV}$, and $N_{\text {j}} = 0$. These requirements are chosen to obtain a ${{\hbox {Z}}_{}^{}} \rightarrow {{\uptau }_{}^{}} ^{+}{{\uptau }_{}^{}} ^{-}$ sample with good purity. The $m({{\upmu }_{}^{}} {{\uptau }_{}^{}} _\mathrm {h} )$ range is chosen to select the ${\hbox {Z}}_{}^{}$ boson peak, low $m_{\mathrm {T}} ({{\upmu }_{}^{}},{\vec p}_{\mathrm {T}}^{\text {miss}})$ helps to remove ${{\hbox {W}}_{}^{}}$+jets and potential signal contamination while the 0-jet requirement helps remove other backgrounds. The $p_{\mathrm {T}} ^\text {miss}$ distribution of these events is shown in Fig. 4 (lower). We observe good agreement between data and the predicted background.

### Estimation of other backgrounds

Smaller contributions are expected from other SM backgrounds, including diboson, triboson, and Higgs boson production. There are also contributions from ${\hbox {t}}_{}^{}$ $\overline{{{\hbox {t}}_{}^{}}}_{}^{}$ and single top quark production, or top quark pair production in association with a vector boson. These are estimated via MC simulation after application of efficiency and energy-scale corrections. Experimental and theoretical uncertainties are evaluated as described below in Sect. 6.

For the $\ell {{\uptau }_{}^{}} _\mathrm {h}$ analyses, we check the BDT distribution in a ${\hbox {t}}_{}^{}$ $\overline{{{\hbox {t}}_{}^{}}}_{}^{}$-enriched CR that is defined by requiring the event selection to be the same as in the SR, except for a requirement of one or two ${\hbox {b}}_{}^{}$-tagged jets. To validate the ${\hbox {W}}_{}^{}$ ${\hbox {W}}_{}^{}$ background prediction, we construct a CR of events with oppositely charged muon-electron pairs that have $m_{{{\upmu }_{}^{}} {{\hbox {e}}_{}^{}}} > 90\,\text {GeV}$ and $N_{\text {j}} = 0$. We obtain systematic uncertainties for the normalization of the corresponding backgrounds and any potential mismodeling of the BDT distribution in these CRs. The latter is done by constructing a $\chi ^2$ test for all CRs with the BDT modeling taken into account by including an additional floating uncertainty that is determined by requiring a *p* value [69] of at least 68% in all CRs. In this way, the BDT shape uncertainty is estimated to be 9%.

## Systematic uncertainties

The dominant uncertainties in this analysis are the statistical uncertainties resulting from limited event counts in data sidebands or in simulated event samples used to obtain background estimates and the systematic uncertainties in the estimated rates for jets to be misidentified as ${{\uptau }_{}^{}} _\mathrm {h}$ candidates. We rely on an extrapolation in ${{\uptau }_{}^{}} _\mathrm {h}$ isolation to obtain an estimate of the background originating from jets misidentified as ${{\uptau }_{}^{}} _\mathrm {h}$ candidates. In the ${{\uptau }_{}^{}} _\mathrm {h} {{\uptau }_{}^{}} _\mathrm {h}$ analysis, the uncertainty in this extrapolation is dominated by the dependence of isolation on jet flavor. It also includes the statistical uncertainty associated with the CR samples from which the extrapolation factors are obtained, which can be significant in the case of search regions with limited event counts that are defined with stringent kinematic requirements. The uncertainty in the combined identification and isolation efficiency for prompt ${{\uptau }_{}^{}} _\mathrm {h}$ candidates is also propagated to the final estimated uncertainty. In the $\ell {{\uptau }_{}^{}} _\mathrm {h}$ analyses, we estimate a transfer factor for the extrapolation in ${{\uptau }_{}^{}} _\mathrm {h}$ isolation from a ${{\hbox {W}}_{}^{}}$+jets-enriched CR. The purity of ${{\hbox {W}}_{}^{}}$+jets events this region is $\approx$85% as determined from simulation. We therefore propagate a relative uncertainty of 15% to account for contamination from other sources.

We use simulation to obtain estimates of the yields from other background contributions and to estimate the potential signal contributions. We propagate uncertainties related to the ${\hbox {b}}_{}^{}$ tagging, trigger, and selection efficiencies, the renormalization and factorization scales, PDFs, jet energy scale and resolution, unclustered energy contributing to $p_{\mathrm {T}} ^\text {miss}$, and the energy scales of electrons, muons, and ${{\uptau }_{}^{}} _\mathrm {h}$ candidates. The correction factors and the corresponding uncertainties for the ${{\uptau }_{}^{}} _\mathrm {h}$ energy scale in simulation are derived from ${{\hbox {Z}}_{}^{}} \rightarrow {{\uptau }_{}^{}} ^{+}{{\uptau }_{}^{}} ^{-}$ events in the $\ell {{\uptau }_{}^{}} _\mathrm {h}$ final states by fits to distributions of the reconstructed ${{\uptau }_{}^{}} _\mathrm {h}$ mass and the visible mass of the $\ell {{\uptau }_{}^{}} _\mathrm {h}$ system [48]. The systematic uncertainties corresponding to energy scale variations can be significant in the ${{\uptau }_{}^{}} _\mathrm {h} {{\uptau }_{}^{}} _\mathrm {h}$ search regions defined with stringent kinematic requirements, which are affected by large statistical uncertainties, because of potentially large event migrations. For the DY+jets background, we have an additional uncertainty associated with the corrections applied to the mass and $p_{\mathrm {T}}$ distributions. We assign a 15% normalization uncertainty in the ${{\uptau }_{}^{}} _\mathrm {h} {{\uptau }_{}^{}} _\mathrm {h}$ final state for the cross sections of processes estimated from simulation, namely DY+jets, ${\hbox {t}}_{}^{}$ $\overline{{{\hbox {t}}_{}^{}}}_{}^{}$, diboson, and rare SM processes, based on the results of CMS differential cross section measurements [70, 71]. For the $\ell {{\uptau }_{}^{}} _\mathrm {h}$ analyses, we extract normalization uncertainties of 5, 5, and 20% for the DY+jets, ${\hbox {t}}_{}^{}$ $\overline{{{\hbox {t}}_{}^{}}}_{}^{}$, and ${\hbox {W}}_{}^{}$ ${\hbox {W}}_{}^{}$ backgrounds, respectively, based on the estimated impurity of the corresponding process-enriched CRs. An additional uncertainty of 9% is assigned to cover potential mismodeling of the BDT distribution in simulation that is based on studies in CRs.

The categorization of events in the ${{\uptau }_{}^{}} _\mathrm {h} {{\uptau }_{}^{}} _\mathrm {h}$ final state by the number of reconstructed jets induces sensitivity to the modeling of ISR in the signal simulation. The $p_{\mathrm {T}} ^{\mathrm {ISR}}$ distribution of simulated signal events is reweighted to improve the ISR modeling. The reweighting factors are obtained from studies of ${\hbox {Z}}_{}^{}$ boson events. We take the deviation of the reweighting factors from unity as a systematic uncertainty.

The uncertainty in the integrated luminosity is taken into account in all background estimates for which we do not extract normalization scale factors in dedicated data CRs, as well as for signal estimates. This uncertainty corresponds to 2.5% [72] and 2.3% [73] for the 2016 and 2017 data, respectively. With the exception of statistical uncertainties, most other uncertainties are of similar size between the 2016 and 2017 analyses. The main systematic uncertainties for signal and background are summarized in Table 2.

In general, we treat all statistical uncertainties as uncorrelated. In addition, all systematic uncertainties arising from statistical limitations in the 2016 and 2017 data are assumed to be uncorrelated while systematic uncertainties from similar sources are treated as correlated or partially correlated across the various background and signal predictions. For the combination of the ${{\uptau }_{}^{}} _\mathrm {h} {{\uptau }_{}^{}} _\mathrm {h}$ and $\ell {{\uptau }_{}^{}} _\mathrm {h}$ analyses, we correlate uncertainties related to object reconstruction, with the exception of the ${{\uptau }_{}^{}} _\mathrm {h}$ selection efficiency, which is treated as uncorrelated because of the use of different isolation algorithms.

**Table 2:** Systematic uncertainties of SM background predictions and a representative signal model, corresponding to a left-handed $\widetilde{\uptau }_{}^{}$, with $m({\widetilde{\uptau }_{}^{}}) = 100\,\text {GeV}$ and $m({{\widetilde{\upchi }}_{1}^{0}}) = 1\,\text {GeV}$. The uncertainty ranges are given in percent. The spread of values reflects uncertainties in different SRs

Uncertainty (%)	Signal	Misidentified ${{\uptau }_{}^{}} _\mathrm {h}$	DY+jets	Top quark	Other SM
${{\uptau }_{}^{}} _\mathrm {h}$ efficiency	5–13	–	5–15	1–14	10–51
${{\hbox {e}}_{}^{}}/{{\upmu }_{}^{}}$ efficiency ($\ell {{\uptau }_{}^{}} _\mathrm {h}$)	2–3	–	2–3	2–3	2–3
${{\uptau }_{}^{}} _\mathrm {h}$ energy scale	0.5–12	–	2.6–27	1.2–11	4.1–13
${{\hbox {e}}_{}^{}}/{{\upmu }_{}^{}}$ energy scale ($\ell {{\uptau }_{}^{}} _\mathrm {h}$)	0.1–25	0.1–5	0.1–30	0.1–20	0.1–10
Jet energy scale	0.5–38	–	1.1–19	0.6–13	2.4–14
Jet energy resolution	0.3–22	–	1.9–10	0.7–22	0.2–11
Unclustered energy	0.3–21	–	2.6–30	0.2–6.4	1.7–14
${\hbox {b}}_{}^{}$ tagging	0.2–0.9	–	0.2–23	1.7–25	0.2–1.2
Pileup	0.9–9.1	–	2–22	0.1–24	0.3–25
BDT distribution ($\ell {{\uptau }_{}^{}} _\mathrm {h}$)	9	–	9	9	9
$\ell \rightarrow {{\uptau }_{}^{}} _\mathrm {h}$ misidentification rate ($\ell {{\uptau }_{}^{}} _\mathrm {h}$)	–	–	–	1	1
Integrated luminosity	2.3–2.5	–	2.3–2.5	2.3–2.5	2.3–2.5
Background normalization	–	10	5–15	2.5–15	15–25
DY+jets mass and $p_{\mathrm {T}}$	–	–	0.2–11	–	–
${{\uptau }_{}^{}} _\mathrm {h}$ misidentification rate	–	4.6–51	–	–	–
Signal ISR	0.2–8.2	–	–	–	–
Renormalization and factorization scales	1.6–7	–	0.7–14	0.7–30	6.7–16
PDFs	–	–	0.1–1.2	0.1–0.4	0.1–0.6

## Results and interpretation

**Fig. 5:**
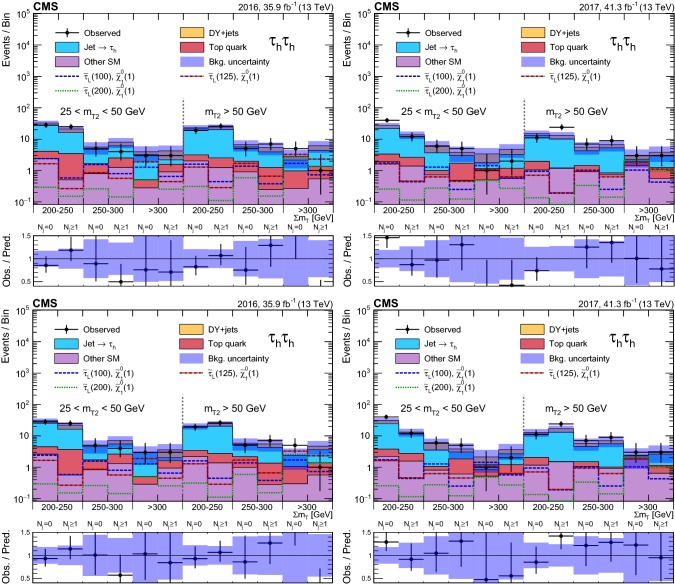
Event counts and predicted yields for the SM background in the ${{\uptau }_{}^{}} _\mathrm {h} {{\uptau }_{}^{}} _\mathrm {h}$ analysis for the 2016 (left) and 2017 (right) data, before (upper) and after (lower) a maximum-likelihood fit to the data. Predicted signal yields are also shown for benchmark signal models of ${\widetilde{\uptau }}_{\mathrm {L}}$ pair production with $m({\widetilde{\uptau }}_{\mathrm {L}})=100$, 125, and 200$\,\text {GeV}$ and $m({{\widetilde{\upchi }}_{1}^{0}})=1\,\text {GeV}$

**Fig. 6:**
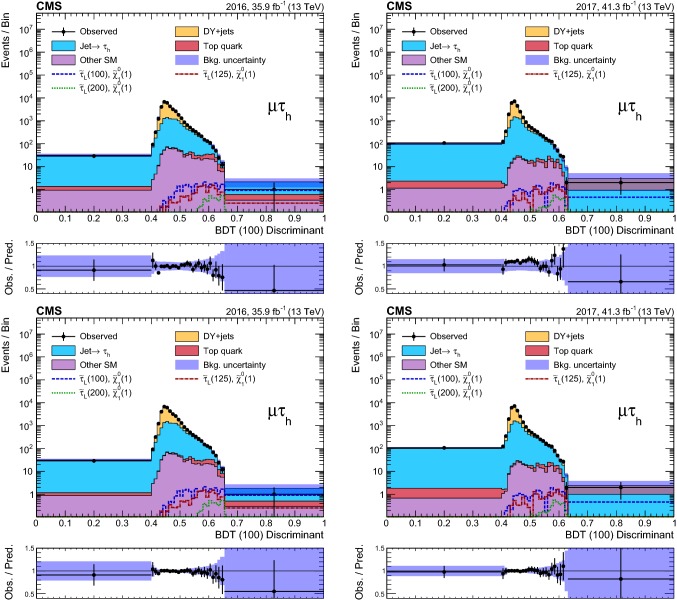
Discriminant distributions for the BDT trained for a $\widetilde{\uptau }_{}^{}$ mass of 100$\,\text {GeV}$ and a ${\widetilde{\upchi }}_{1}^{0}$ mass of 1$\,\text {GeV}$ (BDT (100)) in the ${{\upmu }_{}^{}} {{\uptau }_{}^{}} _\mathrm {h}$ final state for the 2016 (left) and 2017 (right) data, before (upper) and after (lower) a maximum-likelihood fit to the data. Predicted signal yields are also shown for benchmark models of ${\widetilde{\uptau }}_{\mathrm {L}}$ pair production with $m({\widetilde{\uptau }}_{\mathrm {L}})=100$, 125, and 200$\,\text {GeV}$ and $m({{\widetilde{\upchi }}_{1}^{0}})=1\,\text {GeV}$

**Fig. 7:**
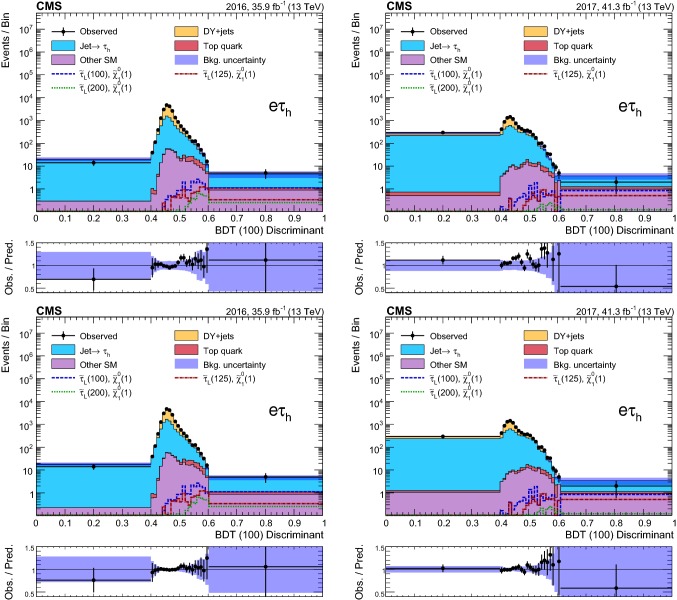
Discriminant distributions for the BDT trained for a $\widetilde{\uptau }_{}^{}$ mass of 100$\,\text {GeV}$ and a ${\widetilde{\upchi }}_{1}^{0}$ mass of 1$\,\text {GeV}$ (BDT (100)) in the ${{\hbox {e}}_{}^{}} {{\uptau }_{}^{}} _\mathrm {h}$ final state for the 2016 (left) and 2017 (right) data, before (upper) and after (lower) a maximum-likelihood fit to the data. Predicted signal yields are also shown for benchmark models of ${\widetilde{\uptau }}_{\mathrm {L}}$ pair production with $m({\widetilde{\uptau }}_{\mathrm {L}})=100$, 125, and 200$\,\text {GeV}$ and $m({{\widetilde{\upchi }}_{1}^{0}})=1\,\text {GeV}$

The results of the search in the ${{\uptau }_{}^{}} _\mathrm {h} {{\uptau }_{}^{}} _\mathrm {h}$ final state are presented in Fig. 5 and summarized in Tables 3 and 4. The background predictions resulting from a maximum likelihood fit to the data under the background-only hypothesis are shown in the lower row of Fig. 5. The BDT distributions corresponding to a training for a $\widetilde{\uptau }_{}^{}$ mass of 100$\,\text {GeV}$ and a ${\widetilde{\upchi }}_{1}^{0}$ mass of 1$\,\text {GeV}$ are shown before and after the maximum-likelihood fit to the data in Figs. 6 and 7 for the ${{\upmu }_{}^{}} {{\uptau }_{}^{}} _\mathrm {h}$ and ${{\hbox {e}}_{}^{}} {{\uptau }_{}^{}} _\mathrm {h}$ final states, respectively. The data are consistent with the prediction for SM background. The predicted and observed event yields in the last, most sensitive BDT bins are summarized in Tables 5 and 6 for $\ell {{\uptau }_{}^{}} _\mathrm {h}$ final states. For the statistical interpretation of these results, the normalization uncertainties affecting background and signal predictions are generally assumed to be log-normally distributed. For statistical uncertainties limited by small event counts in data or simulation, we use a $\varGamma$ distribution.

**Fig. 8:**
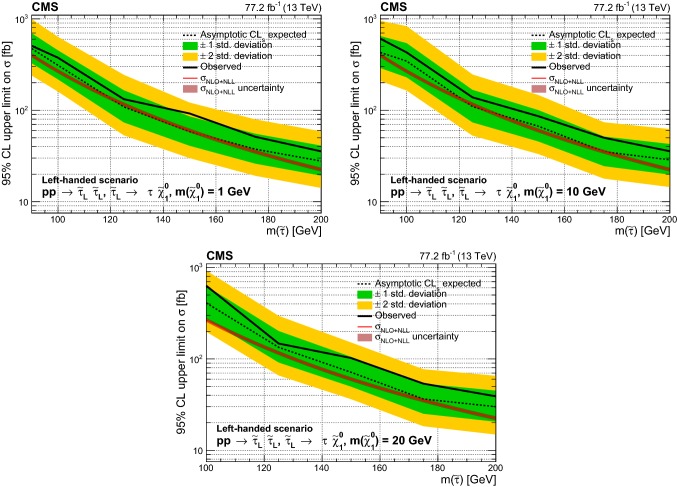
Upper limit on the cross section ($\sigma$) of $\widetilde{\uptau }_{}^{}$ pair production excluded at 95% $\text {CL}$ as a function of the $\widetilde{\uptau }_{}^{}$ mass in the purely left-handed $\widetilde{\uptau }_{}^{}$ models for a ${\widetilde{\upchi }}_{1}^{0}$ mass of 1$\,\text {GeV}$ (upper left), 10$\,\text {GeV}$ (upper right) and 20$\,\text {GeV}$ (lower). The results shown are for the statistical combination of the 2016 and 2017 data in the ${{\uptau }_{}^{}} _\mathrm {h} {{\uptau }_{}^{}} _\mathrm {h}$ and $\ell {{\uptau }_{}^{}} _\mathrm {h}$ analyses. The inner (green) and outer (yellow) bands indicate the respective regions containing 68 and 95% of the distribution of limits expected under the background-only hypothesis. The solid red line indicates the NLO+NLL prediction for the signal production cross section calculated with Resummino [37], while the red shaded band represents the uncertainty in the prediction

**Fig. 9:**
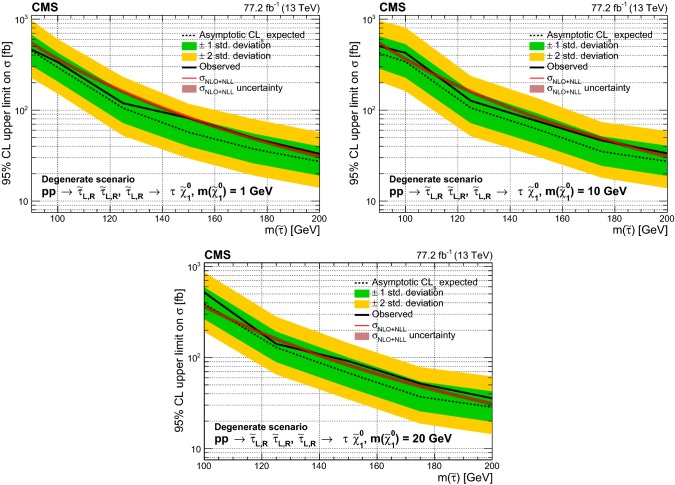
Upper limit on the cross section ($\sigma$) of $\widetilde{\uptau }_{}^{}$ pair production excluded at 95% $\text {CL}$ as a function of the $\widetilde{\uptau }_{}^{}$ mass in the degenerate $\widetilde{\uptau }_{}^{}$ models for a ${\widetilde{\upchi }}_{1}^{0}$ mass of 1$\,\text {GeV}$ (upper left), 10$\,\text {GeV}$ (upper right) and 20$\,\text {GeV}$ (lower). The results shown are for the statistical combination of the 2016 and 2017 data in the ${{\uptau }_{}^{}} _\mathrm {h} {{\uptau }_{}^{}} _\mathrm {h}$ and $\ell {{\uptau }_{}^{}} _\mathrm {h}$ analyses. The inner (green) and outer (yellow) bands indicate the respective regions containing 68 and 95% of the distribution of limits expected under the background-only hypothesis. The solid red line indicates the NLO+NLL prediction for the signal production cross section calculated with Resummino [37], while the red shaded band represents the uncertainty in the prediction

The results are used to set upper limits on the cross section for the production of $\widetilde{\uptau }_{}^{}$ pairs in the context of simplified models [25–27, 74] using all of the exclusive ${{\uptau }_{}^{}} _\mathrm {h} {{\uptau }_{}^{}} _\mathrm {h}$ SRs and the $\ell {{\uptau }_{}^{}} _\mathrm {h}$ BDT distributions in a full statistical combination. The limits are evaluated using likelihood fits with the signal strength, background event yields, and nuisance parameters corresponding to the uncertainties in the signal and background estimates as fitted parameters. The nuisance parameters are constrained within their uncertainties in the fit. We assume that the $\widetilde{\uptau }_{}^{}$ decays with 100% branching fraction to a ${\uptau }_{}^{}$ lepton and a ${\widetilde{\upchi }}_{1}^{0}$. The 95% $\text {CL}$ upper limits on SUSY production cross sections are calculated using a modified frequentist approach with the $\text {CL}_\text {s}$ criterion [75, 76]. An asymptotic approximation is used for the test statistic [77, 78], $q_{\mu } = -2\ln \mathcal {L}_{\mu }/\mathcal {L}_{\text {max}}$, where $\mathcal {L}_{\text {max}}$ is the maximum likelihood determined by allowing all fitted parameters, including the signal strength $\mu$, to vary, and $\mathcal {L}_{\mu }$ is the maximum likelihood for a fixed signal strength. Figure 8 shows the limits obtained for purely left-handed $\widetilde{\uptau }_{}^{}$ pair production, while Fig. 9 shows the limits obtained for the degenerate $\widetilde{\uptau }_{}^{}$ model in which both left- and right-handed $\widetilde{\uptau }_{}^{}$ pairs are produced. The ${{\uptau }_{}^{}} _\mathrm {h} {{\uptau }_{}^{}} _\mathrm {h}$ analysis makes the dominant contribution to the search sensitivity. A slight excess of events over the background expectation in the ${{\uptau }_{}^{}} _\mathrm {h} {{\uptau }_{}^{}} _\mathrm {h}$ SRs results in an observed limit that is weaker than the expected limit. The strongest limits are observed in the case of a nearly massless ${\widetilde{\upchi }}_{1}^{0}$. In general, the constraints are weaker for higher values of the ${\widetilde{\upchi }}_{1}^{0}$ mass because of smaller experimental acceptances. For $\widetilde{\uptau }_{}^{}$ masses above $\approx$150$\,\text {GeV}$, however, the sensitivity does not degrade significantly when the ${\widetilde{\upchi }}_{1}^{0}$ mass increases up to 20$\,\text {GeV}$. In the purely left-handed model, the strongest limits are observed for a $\widetilde{\uptau }_{}^{}$ mass of 125$\,\text {GeV}$ where we exclude a $\widetilde{\uptau }_{}^{}$ pair production cross section of 132$\text { fb}$. This value is a factor of 1.14 larger than the theoretical cross section. In the degenerate $\widetilde{\uptau }_{}^{}$ model we exclude $\widetilde{\uptau }_{}^{}$ masses between 90 and 150$\,\text {GeV}$ under the assumption of a nearly massless ${\widetilde{\upchi }}_{1}^{0}$.

**Table 3:** Predicted background yields and observed event counts in ${{\uptau }_{}^{}} _\mathrm {h} {{\uptau }_{}^{}} _\mathrm {h}$ SRs in 2016 data. For the background estimates with no events in the sideband or in the simulated sample, we calculate the 68% $\text {CL}$ upper limit on the yield. The first and second uncertainties given are statistical and systematic, respectively. We also list the predicted signal yields corresponding to the purely left-handed model for a $\widetilde{\uptau }_{}^{}$ mass of 100$\,\text {GeV}$ and a ${\widetilde{\upchi }}_{1}^{0}$ mass of 1$\,\text {GeV}$

$m_{\mathrm {T2}}$ [$\text {GeV}$ ]	25–50					
$\varSigma m_{\mathrm {T}}$ [$\text {GeV}$ ]	200–250		250–300		>300	
$N_{\text {j}}$	0	$\ge$1	0	$\ge$1	0	$\ge$1
Misidentified ${{\uptau }_{}^{}} _\mathrm {h}$	23.5 ± 2.9 ± 9.8	12.7 ± 2.4 ± 4.2	3.1 ± 1.0 ± 1.7	3.6 ± 1.1 ± 2.0	2.8 ± 0.8 ± 1.8	0.5 ± 0.5 ± 0.2
DY+jets	4.3 ± 2.1 ± 0.7	4.5 ± 1.5 ± 0.9	0.4 ± 0.4 ± 0.1	1.6 ± 0.9 ± 0.3	<0.7	1.5 ± 0.9 ± 0.5
Top quark	1.7 ± 0.3 ± 0.3	2.9 ± 0.4 ± 0.3	0.8 ± 0.2 ± 0.1	1.3 ± 0.2 ± 0.2	0.2 ± 0.1 ± 0.1	0.6 ± 0.2 ± 0.2
Other SM	2.4 ± 0.7 ± 0.4	0.5 ± 0.2 ± 0.1	0.7 ± 0.4 ± 0.1	1.2 ± 0.5 ± 0.3	0.3 ± 0.2 ± 0.1	0.9 ± 0.4 ± 0.2
Total prediction	31.9 ± 3.7 ± 9.8	20.6 ± 2.9 ± 4.3	5.1 ± 1.2 ± 1.7	7.7 ± 1.5 ± 2.1	3.2 ± 0.9 ± 1.8	3.5 ± 1.1 ± 0.6
Observed	28	25	5	4	3	3
$m({\widetilde{\uptau }}_{\mathrm {L}})=100 \,\text {GeV}$	2.4 ± 0.3 ± 0.4	0.6 ± 0.2 ± 0.1	1.6 ± 0.2 ± 0.2	0.8 ± 0.2 ± 0.1	1.3 ± 0.2 ± 0.4	0.7 ± 0.2 ± 0.2

$m_{\mathrm {T2}}$ [$\text {GeV}$ ]	>50					
$\varSigma m_{\mathrm {T}}$ [$\text {GeV}$ ]	200–250		250–300		>300	
$N_{\text {j}}$	0	$\ge$1	0	$\ge$1	0	$\ge$1
Misidentified ${{\uptau }_{}^{}} _\mathrm {h}$	18.2 ± 2.8 ± 9.5	18.1 ± 2.9 ± 6.0	3.7 ± 1.0 ± 2.2	2.7 ± 1.1 ± 0.5	1.1 ± 0.6 ± 0.6	2.9 ± 0.8 ± 1.6
DY+jets	1.1 ± 0.8 ± 0.2	3.3 ± 1.3 ± 0.7	0.5 ± 0.5 ± 0.1	1.0 ± 0.7 ± 0.1	<0.7	1.3 ± 0.8 ± 0.5
Top quark	1.1 ± 0.3 ± 0.1	1.3 ± 0.2 ± 0.3	1.1 ± 0.2 ± 0.2	1.0 ± 0.2 ± 0.1	0.7 ± 0.2 ± 0.1	0.8 ± 0.2 ± 0.1
Other SM	2.0 ± 0.6 ± 0.3	1.2 ± 0.4 ± 0.2	0.9 ± 0.4 ± 0.1	0.2 ± 0.1 ± 0.1	0.3 ± 0.1 ± 0.1	0.5 ± 0.2 ± 0.2
Total prediction	22.5 ± 3.0 ± 9.5	23.9 ± 3.3 ± 6.0	6.2 ± 1.2 ± 2.2	4.9 ± 1.3 ± 0.5	2.1± 0.6 ± 0.6	5.5 ± 1.2 ± 1.7
Observed	19	26	5	7	5	1
$m({\widetilde{\uptau }}_{\mathrm {L}})=100 \,\text {GeV}$	1.6 ± 0.2 ± 0.3	0.4 ± 0.1 ± 0.1	1.4 ± 0.2 ± 0.2	0.4 ± 0.1 ± 0.1	1.7 ± 0.2 ± 0.4	0.7 ± 0.2 ± 0.2

**Table 4:** Predicted background yields and observed event counts in ${{\uptau }_{}^{}} _\mathrm {h} {{\uptau }_{}^{}} _\mathrm {h}$ SRs in 2017 data. For the background estimates with no events in the sideband or in the simulated sample, we calculate the 68% $\text {CL}$ upper limit on the yield. The first and second uncertainties given are statistical and systematic, respectively. We also list the predicted signal yields corresponding to the purely left-handed model for a $\widetilde{\uptau }_{}^{}$ mass of 100$\,\text {GeV}$ and a ${\widetilde{\upchi }}_{1}^{0}$ mass of 1$\,\text {GeV}$

$m_{\mathrm {T2}}$ [$\text {GeV}$ ]	25–50					
$\varSigma m_{\mathrm {T}}$ [$\text {GeV}$ ]	200–250		250–300		>300	
$N_{\text {j}}$	0	$\ge$1	0	$\ge$1	0	$\ge$1
Misidentified ${{\uptau }_{}^{}} _\mathrm {h}$	18.6 ± 3.1 ± 3.6	9.4 ± 2.1 ± 1.7	2.7 ± 0.9 ± 1.0	1.1 ± 0.8 ± 0.3	0.5 ± 0.5 ± 0.1	1.9 ± 0.8 ± 1.3
DY+jets	5.0 ± 2.0 ± 0.7	1.5 ± 0.7 ± 0.2	1.9 ± 1.4 ± 0.5	0.6 ± 0.4 ± 0.2	1.1 ± 0.8 ± 0.3	1.0 ± 0.8 ± 0.1
Top quark	1.2 ± 0.6 ± 0.2	1.1 ± 0.5 ± 0.2	0.2 ± 0.1 ± 0.1	1.0 ± 0.6 ± 0.1	0.3 ± 0.3 ± 0.1	0.5 ± 0.2 ± 0.1
Other SM	1.9 ± 0.7 ± 0.4	1.4 ± 0.6 ± 0.4	0.7 ± 0.5 ± 0.1	0.5 ± 0.5 ± 0.1	0.5 ± 0.3 ± 0.1	0.6 ± 0.4 ± 0.3
Total prediction	26.7 ± 3.8 ± 3.7	13.3 ± 2.3 ± 1.8	5.5 ± 1.8 ± 1.1	3.2 ± 1.2 ± 0.4	2.4 ± 1.0 ± 0.4	4.0 ± 1.2 ± 1.4
Observed	40	12	6	5	1	2
$m({\widetilde{\uptau }}_{\mathrm {L}})=100\,\text {GeV}$	1.7 ± 0.2 ± 0.2	0.4 ± 0.1 ± 0.1	1.3 ± 0.2 ± 0.2	0.3 ± 0.1 ± 0.1	1.4 ± 0.2 ± 0.4	0.6 ± 0.1 ± 0.2

$m_{\mathrm {T2}}$ [$\text {GeV}$ ]	>50					
$\varSigma m_{\mathrm {T}}$ [$\text {GeV}$ ]	200–250		250–300		>300	
$N_{\text {j}}$	0	$\ge$1	0	$\ge$1	0	$\ge$1
Misidentified ${{\uptau }_{}^{}} _\mathrm {h}$	11.2 ± 2.3 ± 4.7	9.0 ± 2.6 ± 1.1	2.8 ± 1.3 ± 0.3	4.5 ± 1.4 ± 1.8	0.2 ± 0.7 ± 0.5	1.6 ± 0.8 ± 0.2
DY+jets	1.3 ± 0.8 ± 0.2	2.6 ± 1.0 ± 0.4	1.0 ± 0.6 ± 0.1	1.0 ± 0.6 ± 0.1	<0.7	0.5 ± 0.5 ± 0.1
Top quark	0.8 ± 0.4 ± 0.1	<0.2	0.3 ± 0.3 ± 0.1	0.1 ± 0.1 ± 0.1	0.4 ± 0.3 ± 0.1	0.6 ± 0.5 ± 0.2
Other SM	1.0 ± 0.4 ± 0.2	1.2 ± 0.6 ± 0.2	0.9 ± 0.5 ± 0.1	0.7 ± 0.5 ± 0.1	1.4 ± 0.7 ± 0.3	0.6 ± 0.4 ± 0.2
Total prediction	14.3 ± 2.5 ± 4.7	12.8 ± 2.8 ± 1.2	5.1 ± 1.5 ± 0.3	6.3 ± 1.6 ± 1.8	2.0 ± 1.0 ± 0.6	3.2 ± 1.1 ± 0.4
Observed	11	24	7	9	3	3
$m({\widetilde{\uptau }}_{\mathrm {L}})=100\,\text {GeV}$	0.9 ± 0.2 ± 0.1	0.2 ± 0.1 ± 0.1	1.0 ± 0.2 ± 0.2	0.3 ± 0.1 ± 0.1	1.0 ± 0.2 ± 0.2	0.4 ± 0.1 ± 0.1

**Table 5:** Predicted background yields and observed event counts in the most sensitive last bins of the BDT distributions in the ${{\hbox {e}}_{}^{}} {{\uptau }_{}^{}} _\mathrm {h}$ and ${{\upmu }_{}^{}} {{\uptau }_{}^{}} _\mathrm {h}$ final states, in data collected in 2016. The numbers in parentheses in the first row are the $\widetilde{\uptau }_{}^{}$ and ${\widetilde{\upchi }}_{1}^{0}$ masses corresponding to the signal model for left-handed $\widetilde{\uptau }_{}^{}$ pair production that is used to train the BDT. In the bottom row, we list the corresponding predicted signal yields in the last bin of the BDT distribution. The first and second uncertainties given are statistical and systematic, respectively

BDT training	BDT(${{\upmu }_{}^{}} {{\uptau }_{}^{}} _\mathrm {h}$,100,1)	BDT(${{\upmu }_{}^{}} {{\uptau }_{}^{}} _\mathrm {h}$,150,1)	BDT(${{\upmu }_{}^{}} {{\uptau }_{}^{}} _\mathrm {h}$,200,1)	BDT(${{\hbox {e}}_{}^{}} {{\uptau }_{}^{}} _\mathrm {h}$,100,1)	BDT(${{\hbox {e}}_{}^{}} {{\uptau }_{}^{}} _\mathrm {h}$,150,1)	BDT(${{\hbox {e}}_{}^{}} {{\uptau }_{}^{}} _\mathrm {h}$,200,1)
Misidentified ${{\uptau }_{}^{}} _\mathrm {h}$	1.6 ± 0.8 ± 0.3	2.3 ± 1.0 ± 0.4	1.5 ± 0.8 ± 0.3	3.3 ± 1.1 ± 0.5	0.2 ± 0.4 ± 0.1	0.5 ± 0.7 ± 0.3
DY+jets	<0.1	0.8 ± 0.8 ± 0.1	<0.1	<0.1	<0.1	0.1 ± 0.1 ± 0.1
Top quark	0.3 ± 0.3 ± 0.1	1.8 ± 1.2 ± 0.2	1.7 ± 1.2 ± 0.6	0.2 ± 0.2 ± 0.1	0.2 ± 0.2 ± 0.1	1.4 ± 0.8 ± 2.0
Other SM	0.3 ± 0.3 ± 0.1	1.4 ± 0.6 ± 0.5	1.5 ± 0.6 ± 0.4	0.9 ± 0.5 ± 0.4	0.6 ± 0.4 ± 0.5	2.0 ± 0.7 ± 1.0
Total prediction	2.1 ± 0.9 ± 0.4	6.4 ± 1.8 ± 1.0	4.6 ± 1.6 ± 0.9	4.5 ± 1.3 ± 0.8	1.0 ± 0.6 ± 0.5	4.2 ± 1.3 ± 1.8
Observed	1	6	7	5	2	7
Signal	1.3 ± 0.4 ± 0.2	0.9 ± 0.2 ± 0.1	0.7 ± 0.1 ± 0.5	1.5 ± 0.4 ± 0.2	0.4 ± 0.1 ± 0.1	1.0 ± 0.1 ± 0.2

**Table 6:** Predicted background yields and observed event counts in the most sensitive last bins of the BDT distributions in the ${{\hbox {e}}_{}^{}} {{\uptau }_{}^{}} _\mathrm {h}$ and ${{\upmu }_{}^{}} {{\uptau }_{}^{}} _\mathrm {h}$ final states, in data collected in 2017. The numbers in parentheses in the first row are the $\widetilde{\uptau }_{}^{}$ and ${\widetilde{\upchi }}_{1}^{0}$ masses corresponding to the signal model for left-handed $\widetilde{\uptau }_{}^{}$ pair production that is used to train the BDT. In the bottom row, we list the corresponding predicted signal yields in the last bin of the BDT distribution. The first and second uncertainties given are statistical and systematic, respectively

BDT training	BDT(${{\upmu }_{}^{}} {{\uptau }_{}^{}} _\mathrm {h}$,100,1)	BDT(${{\upmu }_{}^{}} {{\uptau }_{}^{}} _\mathrm {h}$,150,1)	BDT(${{\upmu }_{}^{}} {{\uptau }_{}^{}} _\mathrm {h}$,200,1)	BDT(${{\hbox {e}}_{}^{}} {{\uptau }_{}^{}} _\mathrm {h}$,100,1)	BDT(${{\hbox {e}}_{}^{}} {{\uptau }_{}^{}} _\mathrm {h}$,150,1)	BDT(${{\hbox {e}}_{}^{}} {{\uptau }_{}^{}} _\mathrm {h}$,200,1)
Misidentified ${{\uptau }_{}^{}} _\mathrm {h}$	0.9 ± 0.5 ± 0.4	<0.1	<0.1	2.5 ± 0.9 ± 1.3	0.3 ± 0.3 ± 0.1	<0.1
DY+jets	2.1 ± 2.1 ± 3.3	<0.1	<0.1	<0.1	<0.1	<0.1
Top quark	<0.1	0.9 ± 0.4 ± 0.8	0.6 ± 0.5 ± 0.5	0.3 ± 0.3 ± 0.1	<0.1	0.2 ± 0.2 ± 0.2
Other SM	<0.1	1.0 ± 0.7 ± 1.6	0.6 ± 0.6 ± 1.1	1.0 ± 0.7 ± 1.5	0.2 ± 0.2 ± 0.5	1.0 ± 0.6 ± 1.6
Total prediction	3.0 ± 2.2 ± 3.1	2.0 ± 1.0 ± 2.0	1.2 ± 0.7 ± 1.3	3.7 ± 1.1 ± 2.3	0.4 ± 0.4 ± 0.5	1.2 ± 0.7 ± 1.6
Observed	2	6	2	2	1	1
Signal	0.6 ± 0.3 ± 0.1	0.4 ± 0.1 ± 0.8	0.6 ± 0.1 ± 0.3	1.0 ± 0.4 ± 0.1	0.2 ± 0.1 ± 0.1	0.2 ± 0.1 ± 0.1

## Summary

A search for direct ${\uptau }_{}^{}$ slepton ($\widetilde{\uptau }_{}^{}$) pair production has been performed in proton–proton collisions at a center-of-mass energy of 13$\,\text {TeV}$ in events with a ${\uptau }_{}^{}$ lepton pair and significant missing transverse momentum. Search regions are defined using kinematic observables that exploit expected differences in discriminants between signal and background. The data used for this search correspond to an integrated luminosity of 77.2$\,\text {fb}^{-1}$ collected in 2016 and 2017 with the CMS detector. No excess above the expected standard model background has been observed. Upper limits have been set on the cross section for direct $\widetilde{\uptau }_{}^{}$ pair production for simplified models in which each $\widetilde{\uptau }_{}^{}$ decays to a ${\uptau }_{}^{}$ lepton and the lightest neutralino, with the latter being assumed to be the lightest supersymmetric particle. For purely left-handed $\widetilde{\uptau }_{}^{}$ pair production, the analysis is most sensitive to a $\widetilde{\uptau }_{}^{}$ mass of 125$\,\text {GeV}$ when the neutralino is nearly massless. The observed limit is a factor of 1.14 larger than the expected production cross section in this model. The limits observed for left-handed $\widetilde{\uptau }_{}^{}$ pair production are the strongest obtained thus far for low values of the $\widetilde{\uptau }_{}^{}$ mass. In a more optimistic, degenerate production model, in which both left- and right-handed $\widetilde{\uptau }_{}^{}$ pairs are produced, we exclude $\widetilde{\uptau }_{}^{}$ masses up to 150$\,\text {GeV}$, again under the assumption of a nearly massless neutralino. These results represent the first exclusion reported for this model for low values of the $\widetilde{\uptau }_{}^{}$ mass between 90 and 120$\,\text {GeV}$.

## Data Availability

This manuscript has no associated data or the data will not be deposited. [Authors’ comment: Release and preservation of data used by the CMS Collaboration as the basis for publications is guided by the CMS policy as written in its document “CMS data preservation, re-use and open access policy” (https://cms-docdb.cern.ch/cgi-bin/PublicDocDB/RetrieveFile?docid=6032&filename=CMSDataPolicyV1.2.pdf&version=2).]
